# Aromatic L-Amino Acid Decarboxylase (AADC) Is Crucial for Brain Development and Motor Functions

**DOI:** 10.1371/journal.pone.0071741

**Published:** 2013-08-05

**Authors:** De-Fen Shih, Chung-Der Hsiao, Ming-Yuan Min, Wen-Sung Lai, Chianne-Wen Yang, Wang-Tso Lee, Shyh-Jye Lee

**Affiliations:** 1 Institute of Zoology, National Taiwan University, Taipei, Taiwan, R.O.C; 2 Department of Bioscience Technology, Chung Yuan Christian University, Chung-Li, Taiwan, R.O.C; 3 Department of Life Science, National Taiwan University, Taipei, Taiwan, R.O.C; 4 Department of Psychology, National Taiwan University, Taipei, Taiwan, R.O.C; 5 Department of Pediatrics, National Taiwan University Hospital, Taipei, Taiwan, R.O.C; 6 Clinical Center for Neurosciences and Behavioral Medicine, National Taiwan University Hospital, Taipei, Taiwan, R.O.C; 7 Graduate Institute of Brain and Mind Sciences, National Taiwan University, Taipei, Taiwan, R.O.C; 8 Center for Biotechnology, National Taiwan University, Taipei, Taiwan, R.O.C; 9 Research Center for Developmental Biology and Regenerative Medicine, National Taiwan University, Taipei, Taiwan, R.O.C; University of Utah School of Medicine, United States of America

## Abstract

Aromatic L-amino acid decarboxylase (AADC) deficiency is a rare pediatric neuro-metabolic disease in children. Due to the lack of an animal model, its pathogenetic mechanism is poorly understood. To study the role of AADC in brain development, a zebrafish model of AADC deficiency was generated. We identified an *aadc* gene homolog, *dopa decarboxylase* (*ddc*), in the zebrafish genome. Whole-mount *in situ* hybridization analysis showed that the *ddc* gene is expressed in the epiphysis, locus caeruleus, diencephalic catecholaminergic clusters, and raphe nuclei of 36-h post-fertilization (hpf) zebrafish embryos. Inhibition of Ddc by AADC inhibitor NSD-1015 or anti-sense morpholino oligonucleotides (MO) reduced brain volume and body length. We observed increased brain cell apoptosis and loss of dipencephalic catecholaminergic cluster neurons in *ddc* morphants (*ddc* MO-injected embryos). Seizure-like activity was also detected in *ddc* morphants in a dose-dependent manner. *ddc* morphants had less sensitive touch response and impaired swimming activity that could be rescued by injection of *ddc* plasmids. In addition, eye movement was also significantly impaired in *ddc* morphants. Collectively, loss of Ddc appears to result in similar phenotypes as that of ADCC deficiency, thus zebrafish could be a good model for investigating pathogenetic mechanisms of AADC deficiency in children.

## Introduction

Aromatic L-amino acid decarboxylase (AADC) deficiency is an important neuro-metabolic disease in children. Children with AADC deficiency usually present with severe developmental delay, oculogyric crises (an eyeball movement disorder), generalized hypotonia, paroxysmal dystonia, and autonomic dysfunction [1–7]. After the first report of AADC deficiency in 1990 [8], many cases have been diagnosed and detail descriptions of disease symptoms have been presented [1–7]. Although the clinical features and management have been delineated, the pathogenetic mechanism of AADC deficiency and its role in brain development remain unclear [1,3–6,9,10].

Moreover, AADC is responsible for the decarboxylation step in the catecholamine and dopamine biosynthesis. Dopamine and serotonin can be synthesized by AADC from L-3,4-dihydroxyphenylalanine and 5-hydroxytryptophan, respectively [7]. A deficiency in AADC will lead to reduced biogenic monoamines, including dopamine, norepinephrine, epinephrine, and serotonin (Hyland et al., 1992; Swoboda et al., 1999). The characteristic pattern of abnormalities in cerebrospinal fluid in patients with AADC deficiency includes low homovanillic acid (HVA) and 5-hydroxyindoleacetic acid (5-HIAA) levels, and elevated L-3,4-dihydroxyphenylalanine (L-DOPA) and 3-o-methyldopa levels.

The main neuro-modulators in the brain and spinal cord can govern mood regulation, cognitive and physiological homeostasis, and motor coordination [11–14]. Adequate stimulation of dopamine, serotonin, and adrenergic receptors in specific developmental stages of the brain is important for normal motor and cognitive development [15,16]. Furthermore, dopamine plays important roles in modulating neuronal functions. There are three main brain dopaminergic pathways: the nigro-striatal pathway, the meso-limbic pathway, and the meso-cortical pathway. Impairment of dopamine metabolism in AADC deficiency may therefore lead to motor and cognitive dysfunction. Aside from dopamine, serotonin and norepinephrine may also affect neuronal development [16]. Altered serotonergic and adrenergic functions may be related to some psychiatric conditions seen in patients with AADC deficiency. Moreover, AADC is functionally associated with several neurologic disorders, including Parkinson’s disease [17,18], and may therefore play an important role in brain development.

The function of AADC has been well studied in different species in adrenal, brain, kidney, intestine, liver, and lung tissues [19–29]. Deficiency in AADC co-factor, pyridoxine, causes reduced body weight and liver function in post-natal rats [30]. Monoamine oxidase-B inhibitor can also down-regulate AADC and impair motility in rhesus monkey [31].

However, despite the lower level of AADC expression in brain [32], the pathogenic mechanisms of neurological defects observed in AADC deficiency remains unclear due to limited studies. Because patients with AADC deficiency frequently have prominent brain atrophy and hypo-myelination [4,6], establishing a reliable animal model to screen for potential treatment and investigation possible pathogenic mechanisms is mandatory.

Zebrafish (*Danio rerio*) is an emerging vertebrate model with numerous advantages for gene functional assay [33–35]. A functional nervous system is established within 4-5 days of embryonic development that makes complex behavioral assays like swimming and hunting behaviors possible [36,37]. The dopamine (DA) neuronal system is well described in both larvae [38] and adult fish [39]. In zebrafish, DA neurons are first detected at 18 h post-fertilization (hpf) in a group of cells in the posterior tuberculum of the ventral diencephalon [40]. Such neurons are exclusively located in the forebrain, with majority of groups in the ventral diencephalon located in the ventral thalamus, posterior tuberculum, and hypothalamus, and ascending to the subpallium, comparable to the human nigro-striatal system [41,42].

In this study, we identified an *aadc* gene homolog, *dopa decarboxylase* (*ddc*), in the zebrafish genome. The nucleotide and amino acid sequences in zebrafish Ddc were highly conserved compared to its human and rat homologs. The inhibition of *ddc* expression or activity in zebrafish embryos resulted in defects in morphology and behaviors as phenotypes observed in human AADC deficiency. Zebrafish may thus serve as an important model system for AADC deficiency research.

## Results

### Identification and expression of zebrafish *ddc*

**Figure 1:**
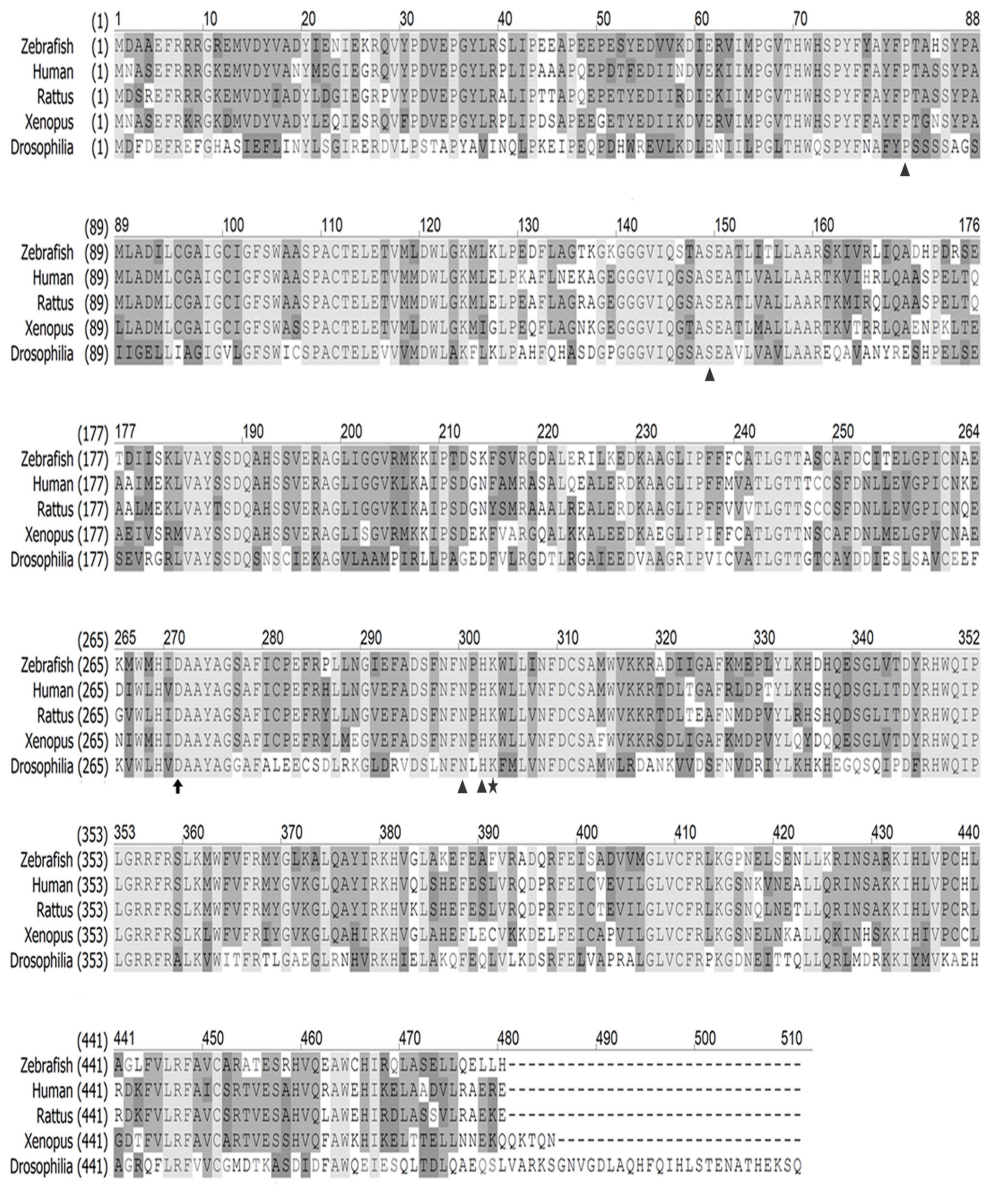
Alignment analysis of AADC among vertebrates. AADC protein was highly conserved with other species. The amino acid sequence was similar when comparing AADC among zebrafish, human, rat, African clawed frog and fruit fly. Sequence alignment was done using the software ClustaW2. Arrow, Asp 271 as salt bridge; arrowheads, active site; star, PLP binding site.

A zebrafish *ddc* gene was cloned according to an NCBI reference sequence (NM_213342.1) and aligned it with homologs from human, rat, fruit fly, and African clawed frog using the CLUSTALW2 software (http://www.ebi.ac.uk/Tools/msa/clustalw2/) (Figure 1). The zebrafish *ddc* gene encodes a 480-amino acid monomeric protein. The zebrafish Ddc protein functional residues are conserved as that of its mammalian orthologs. The putative active site residues, including Thr82, Ser149, Asn300, and His302 (arrow heads), are located in the interface between two monomers, whereas Ile101and Phe103 provided by the adjacent monomer are used for binding with the other subunits [43]. An AADC co-factor, pyridoxal phosphate (PLP), is bound to Lys303 (star) to form an internal Schiff base linkage while another residue Asp271 (arrow) is crafted as a salt bridge through PLP.

All residues mentioned above are conserved among all species examined (Figure 1). The zebrafish *ddc* gene is highly conserved with 88% identity to its human homolog and it is closer to mammalian AADCs compared to other animal species phylogenetically (data not shown).

**Figure 2:**
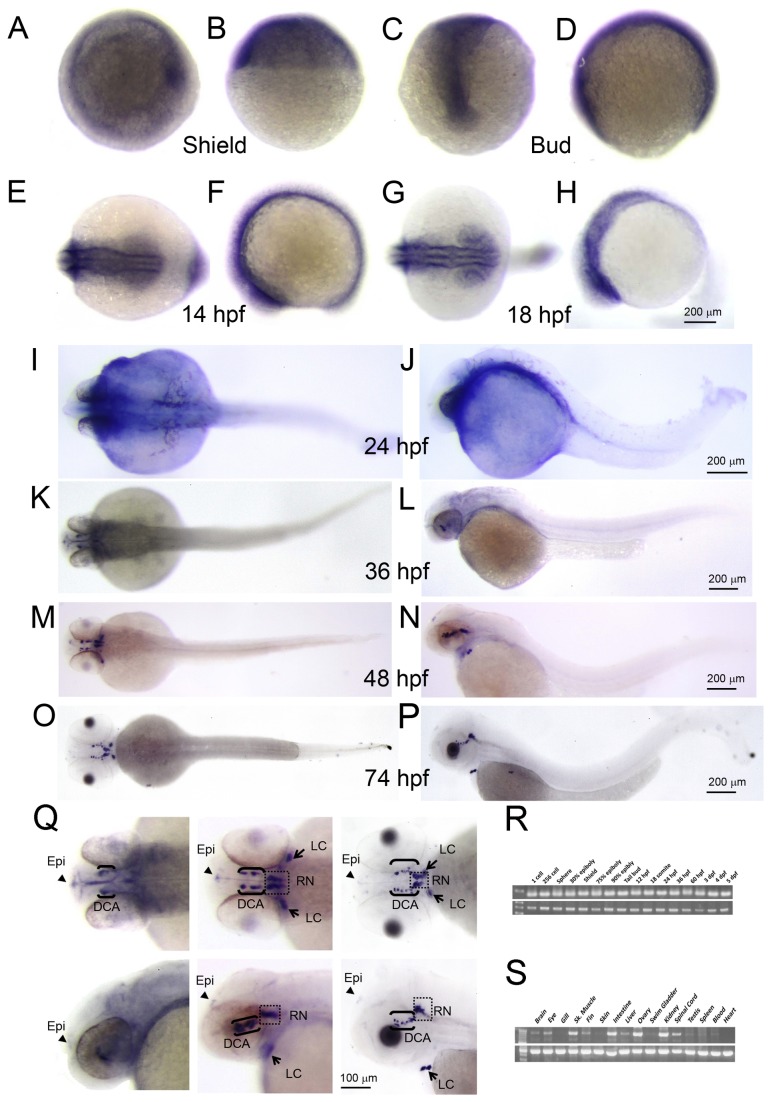
*ddc* expression pattern in zebrafish. *ddc* mRNA was expressed from ubiquitous to specific areas in brain during whole developmental stage. Whole mount RNA in situ hybridization was performed using a *ddc-*specific anti-sense probe on embryos at (**A**–**B**) shield stage, (**C**–**D**) bud stage, (**E**–**F**) 14 hpf, (**G**–**H**) 18 hpf, (**I**–**J**) 24 hpf, (**K**–**L**) 36 hpf, (**M**–**N**) 48 hpf, and (**O**–**P**) 74 hpf. (**Q**) was amplified from 36 hpf, 48 hpf, and 74 hpf, respectively. Panels (**A**)**, **(**C**)**, **(**E**)**, **(**G**)**, **(**I**)**, **(**K**)**, **(**M**) and (**O**) are dorsal view. The rest are lateral view. Arrowheads, epiphysis (Epi); arrows, direct locus coeruleus (LC); brackets, diencephalic catecholaminergic cluster (DCA); staining in square, raphe nuclei (RN). (**R**–**S**) The RT-PCR analysis for *ddc* expression in time scan and Adult tissue scan.

Zebrafish central nervous system is developed from the bud stage and matured at 4-5 days post fertilization (dpf). During central nervous system development, zebrafish DA neurons branch out from the ventral region of an embryo at the 18-somite stage. To investigate the *ddc* expression in the central nervous system during development, a truncated (500 bps) and a full length (1450 bps) zebrafish *ddc* anti-sense RNA probes were synthesized. Whole-mount *in situ* hybridization of both probes showed essentially the same patterns, so we only show results using the 500-bp probes for the shield, bud, 12-somite, 18-somite, 24, 36, 48, and 74 hpf stage embryos (Figure 2A–2Q). All experiments were repeated three times with over 30 larvae used in each experiment.

During early embryogenesis (shield to bud stage), the *ddc* gene was ubiquitously expressed (Figure 2A–2D). The expression domains later became more restricted to the ventral side of central nervous system. After 18 hpf, the *ddc* expression domain was only in the ventral region of the central nervous system (Figure 2G-2H). At 48 hpf and 74 hpf, the *ddc* expression domains were specifically expressed in DA and serotoninergic neurons of the ventral diencephalon (Figure 2 M–2Q). The *ddc* was expressed in the locus caeruleus (arrows), which can synthesize norepinephrine, diencephalic catecholaminergic (DCA) cluster (brackets), where the DA neurons are located, and in the raphe nuclei (square), which can release serotonin (Figure 2Q).

In addition, the *ddc* gene was expressed in the epiphysis as in the pineal gland in human brain after the 36-hpf stage (Figure 2Q, arrowhead). However, there was only a slight expression in the spinal cord, which was in contrast to a notable *ddc* expression in adult spinal cord, as shown by RT-PCR analysis (Figure 2R). The *ddc* gene was also expressed in the central nervous system, digestive system, muscle, and ovary (Figure 2R) and was expressed from 1-cell to 5 dpf-stage embryos (Figure 2S).

### Inhibition or down-regulation of Ddc causes developmental and behavioral defects

**Figure 3:**
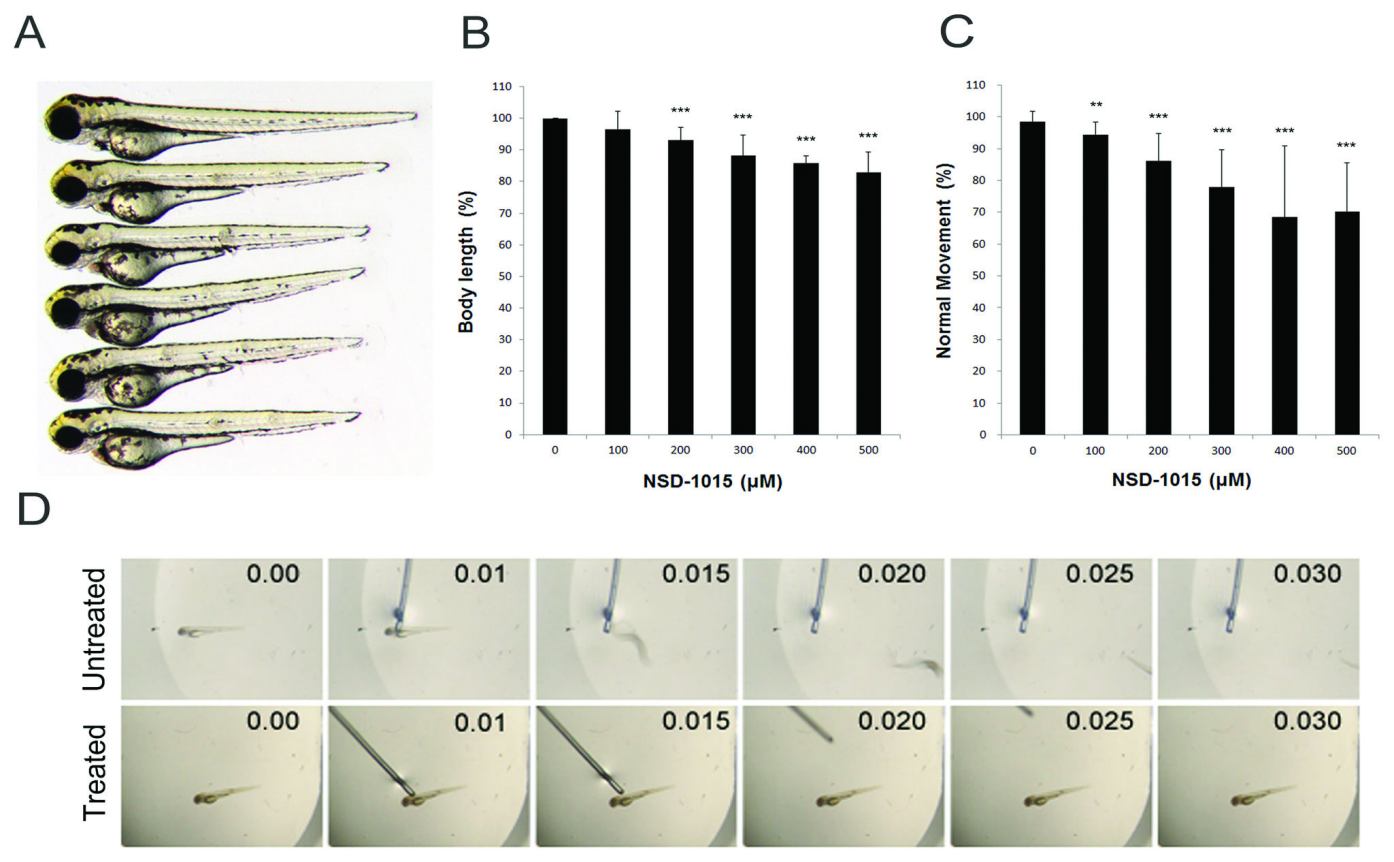
Inhibition of AADC by NSD-1015 in zebrafish. AADC inhibitor NSD-1015 caused morphological and behavioral defects. Compared to untreated fish, the inhibited larvae became shorter by gradient increase of NSD-1015 concentration. (**A**) The phenotype of treated larvae. From up to bottom is the untreated larvae (n=234), and treated larvae by inhibitor NSD-1015 100 µM(n= 312), 200 µM (n=300), 300 µM (n=304), 400 µM (n=264), and 500 µM (n=260). (**B**–**C**) The statistics of body length and movement in touch response, respectively, and showed the decrease of body length and movement in touch response in larvae treated with NSD-1015. (**D**) Sequencing touch response for untreated (upper panel) and treated larvae (bottom panel), showing decreased movement in touch response in larvae treated with NSD-1015. Results were expressed as mean±SD. **p<0.01, ***p<0.001 compared to control larvae.

To investigate the function of Ddc in zebrafish, both pharmaceutical inhibitor and anti-sense MOs were used to decrease AADC activity. NSD-1015 (3-hydroxybenzylhydrazine dihydrochloride) is a commonly-used AADC inhibitor that can specifically inhibit the decarboxylation activity of AADC [44]. Table 1 shows the effective inhibition of NSD-1015 on zebrafish AADC activity by treating zebrafish protein lysate with NSD-1105. To avoid interference on early embryogenesis, dechorionated zebrafish embryos were treated with 100-500 µM NSD-1015 after the bud stage. The NSD-1015 treatment caused dose-dependent shortening of body length in 3-dpf larvae (Figure 3A). The shortening of body length was from 96.4% (100 µM, n=312) to 83% (500 µM, n=260) of control larvae (Figure 3B).

**Table 1:** AADC activity assays.

**Treatment**	**AADC-activity (pmole/h/mg protein)**
NSD-1015 (μM)	
0	10.9 ± 1.6
500	3.5±1.9
tMO1 (ng)	
0	14.0 ± 1.1
2.5	ND
5	ND

ND : not detectable

Zebrafish embryos at 1-cell stage were microinjected without or with designated amount of *ddc* tMO1, cultured to 3 dpf and subjected to AADC activity assay in the presence or absence of NSD-1015 as described in the Materials and Methods.

Touch response assay was also performed to examine the effect of NSD-1015 on larval motility. Poking on the trigeminal neuron posterior to the eyes resulted in a rapid escaping response in untreated control larvae (Supplementary Movie S1). In contrast, the escaping behavior was dose-dependently inhibited by NSD-1015 in treated larvae (Supplementary Movie S2, Figure 3C). A series of snapshots from a 30-sec recording for a larva treated without or with NSD-1015 are presented (Figure 3D).

To specifically block the *ddc* gene activity, two anti-sense MOs (designated tMO1 and tMO2, sequence shown in Supplementary Table S1) were used to inhibit Ddc translation. The tMOs efficiency and specificity were examined by co-injecting a pCS2^+^ vector harboring the respective *ddc* tMO targeting binding sequences (Figure S1A). Both tMOs effectively blocked the expression of green fluorescent protein (GFP) (Figure S1B& C).

To confirm the inhibition of tMO on endogenous AADC activity in zebrafish embryos, AADC activity assay was conducted. The AADC activity was clearly present in protein lysates collected from untreated and sham-injected embryos. In contrast, the AADC activity was under the detection level in tMO1-treated embryos (Table 1). The MO-injected embryos were called morphants thereafter.

**Figure 4:**
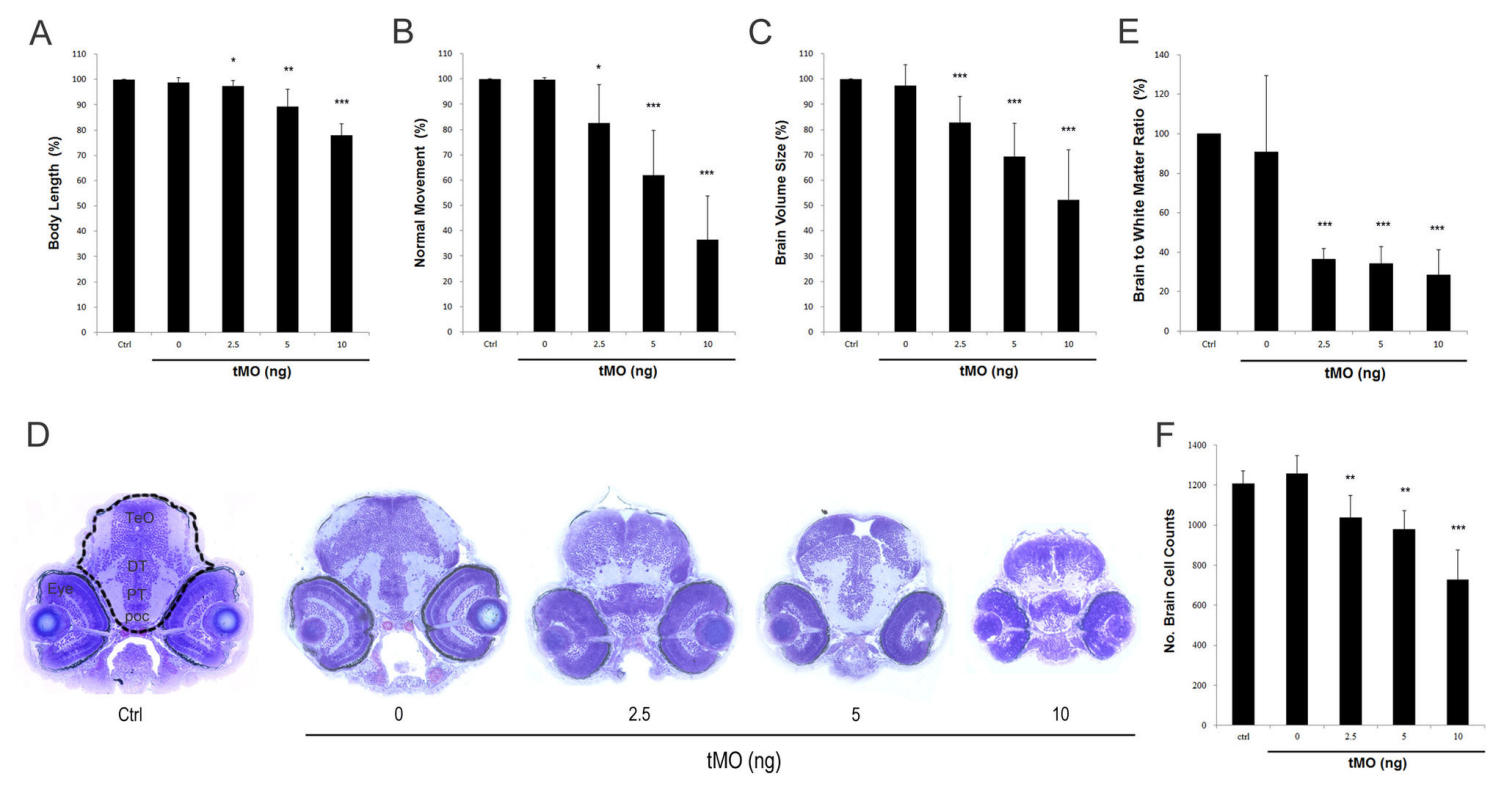
Morphologic and behavioral changes observed in *ddc* morphants. The change of body length (**A**) and touch response (**B**) in control (n=124) and 0 (n=123), 1.25 ng (n=104), 2.5 ng (n=93), 5 ng (n=111) and 10 ng (n=96) tMO1-treated larvae, showing the dose-dependent decrease of body length and touch response in treated larvae. (**C**) Brain volume calculation showing the decrease of brain volume compared with control, in 1.25, 2.5, 5 and 10 ng tMO1-treated larvae (n=7 in each test). (**D**) Compared with control and buffer image stained with Toluidine blue, horizontal section in 3 dpf morphants revealed smaller brain size in 2.5, 5 and 10 ng morphants. DT, dosal thalamus; poc, postoptic commissure; PT, posterior tuberculum; TeO, tectum opticum. Statistical analyses of brain to white matter ratio (**E**) and brain cell numbers (**F**) in control, and 0, 1.25, 2.5, 5 and 10 (n=7 each) ng tMO1-treated larvae are shown. The brain area is enclosed by a dash line boundary and the white mater area is light blue staining areas within the brain. All results were expressed as mean±SD. *p<0.05, **p<0.01, ***p<0.001 compared to control larvae.

The *ddc* morphants showed similar defects like that of NSD-1015-treated embryos. Compared with control larvae, significant reductions in body length to 89.2% (n=111) and 77.9% (n=96) were observed in 5 and 10 ng tMO1-treated embryos, respectively (Figure 4A). There was a more dramatic inhibition on touch response in *ddc* morphants. At 10 ng, only 36.5±17.3% (n=94) of *ddc* morphants showed normal touch escaping response (Figure 4B). To determine the effect of *ddc* tMO1 on brain development, 3-dpf control embryos and *ddc* morphants were fixed and subjected to plastic sections, as described previously [45]. Toluidine blue stain revealed that brain chamber was notably reduced in *ddc* morphants (Figure 4D). By measuring and calculating the brain chamber volume in each section, the total brain volume was summed from 10 sections. The brain volume was significantly reduced to 82.9±10.3%, 69.3±13.1% and 52.3±19.8% in 2.5, 5, and 10 ng tMO1-treated groups, respectively (n=7, *p*<0.05). Whole-mount *in situ* hybridization against HuC, a known neuronal marker, further confirmed the reduction of brain in *ddc* morphants (Figure S2). In addition, to observe the change in brain structure, the ratio of brain to white matter were calculated in *ddc* morphants and untreated control embryos. Compared to that of control, there was a significant decrease in brain to white matter ratios to 36.4±5.5%, 34.0±6.9% and 28.3±13.1% in 2.5, 5, and 10 ng tMO1-treated embryos, respectively (Figure 4E, n=7).

Figure 3A shows a clear reduction of head size in the NSD-1015-treated larvae. The *ddc* morphants also exhibited similar phenotype (data not shown). To further clarify the pathogenetic mechanisms of brain volume reduction in morphants, the total cell numbers in selected brain sections were counted using the ImageJ Software. Compared to the control larvae (1207.9±62.5), brain cell number significantly decreased to 1037.6±110.4, 978.9±93.1, and 726.3±150.2 per section in 2.5, 5, and 10 ng tMO1-treated morphants, respectively (Figure 4F, n=7).

**Figure 5:**
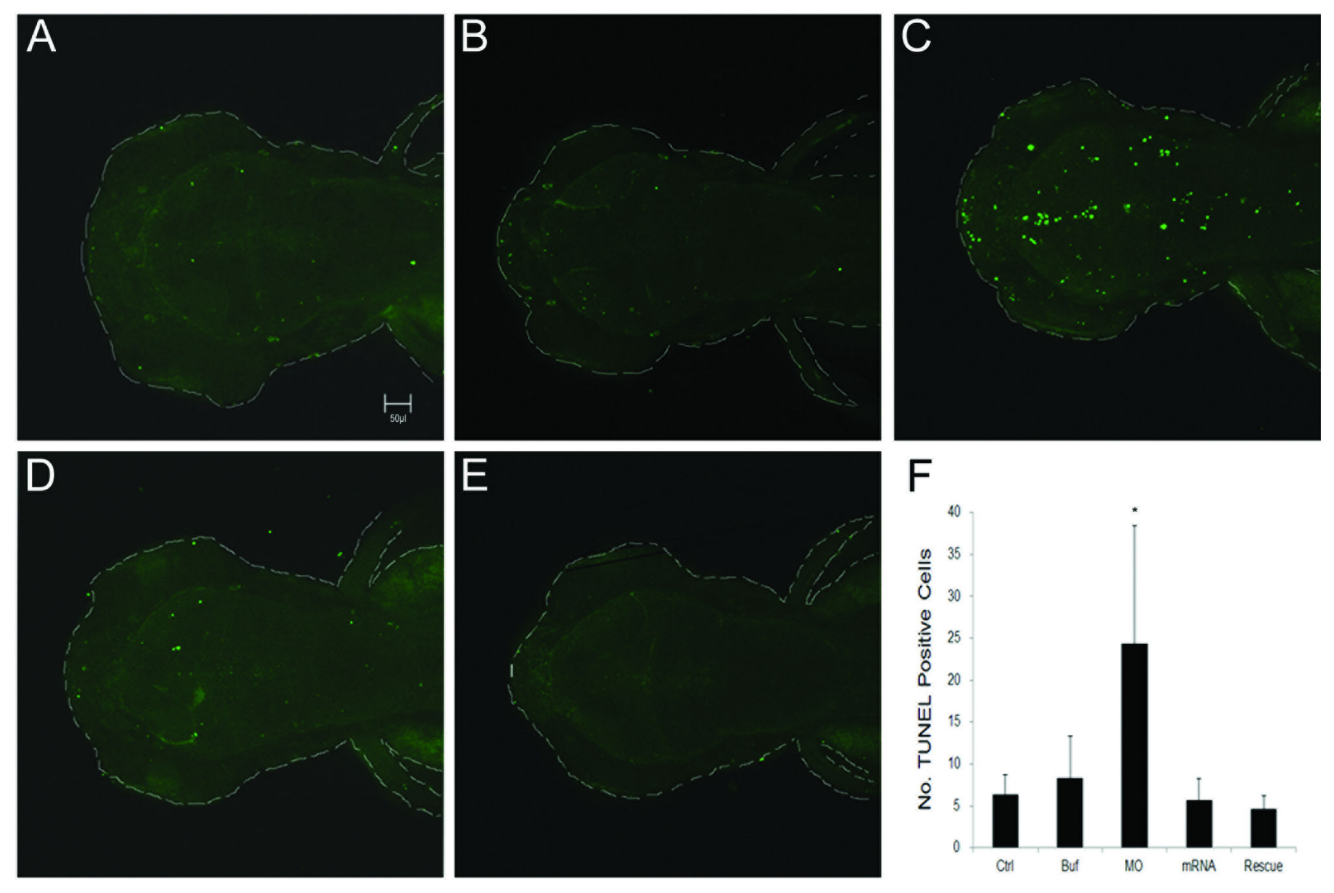
TUNEL staining for apoptosis in treated larvae. All larvae were evaluated at 3 dpf. (**A**) Control. (**B**) Injection of 1x Danieaus’ buffer with 1% phenol red. (**C**) Injection of 5 ng *ddc* tMO1. (**D**) Injection of 100 pg *ddc* mRNA. (**E**) Injection of mixture of 5 ng *ddc* tMO1 and 50 pg *ddc* mRNA with non-MO1 binding site. Apoptotic cells were increased in *ddc* morphants compared to other controls. The increase of apoptosis can be attenuated by co-injection of *ddc* mRNA. (F) Statistical analysis. Photographs were taken by Zeiss Confocal 700 microscope at 100X magnification. All data represented at least three independent experiments and were expressed as mean±SD. *p<0.05 compared to control larvae.

To determine whether inhibition of AADC activity could enhance apoptosis, TUNEL staining was performed to examine cell apoptosis during development. Enhanced cell apoptosis was observed in brain and trunk regions of *ddc* morphants compared to that of control embryos. To examine the effect of *ddc* MO on brain development, we focused on the brain region. *ddc* morphants showed increased apoptotic cells (24.4±14.0) in peri-ventricular areas of brain compared to that of control embryos (6.4±2.3) (Figure 5A, n=16). It could be significantly rescued by co-injection of *ddc* mRNA with mutations at its tMO1 binding site to *ddc* morphants for rescue (4.7±1.6, *p*<0.05) (Figure 5E). As a control, injection of the mutated *ddc* mRNA alone did not notably affect apoptosis (5.7±2.6, n=16, Figure 5D). Quantitative analysis showed a 4-fold increase in apoptotic cells in *ddc* morphants compared to that of control embryos (Figure 5F).

### Loss of Ddc interferes DA neuron patterning

**Figure 6:**
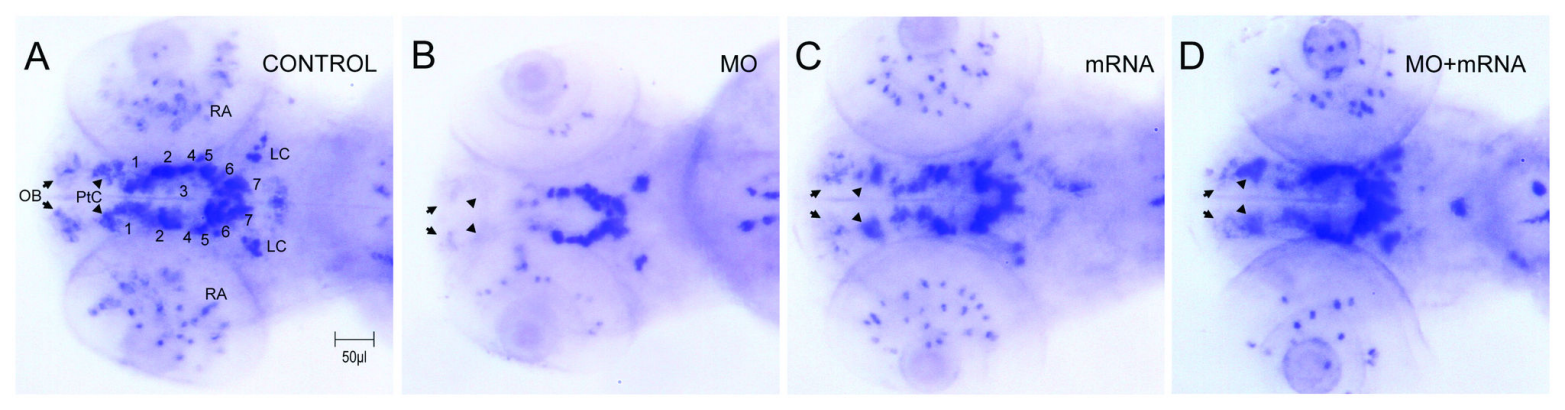
Morphological changes in DA neuron patterning. In situ hybridization with tyrosine hydroxylase anti-sense mRNA probe. (**A**) Control. (**B**) 5 ng *ddc* tMO1 treated. (**C**) 100 pg *ddc* mRNA treated. (**D**) Co-injected mixture with 5 ng *ddc* tMO1 and 50 pg *ddc* mRNA with non-tMO1 binding site. Numbers show the site of dopaminergic neuron clusters 1-7 and 8, which indicated spots in retina, locus coeruleus (LC), pre-tectum (PrC, arrowhead) and olfactory bulb (OB, arrow). Compared to tyrosine hydroxylase expression pattern in control larvae, several tissue parties including olfactory bulb, pre-tectum and DA cluster were malpositioned or absent in the *ddc* morphant brains.

To further investigate the dopaminergic neurons in *ddc* knockdown larvae, we used tyrosine hydroxylase (TH) as a marker for dopaminergic neurons [38,46]. As shown in previous reports [46,47], we showed that the tyrosine hydroxylase is expressed in catecholaminergic clusters and raphe nuclei in untreated control (Figure 6A) and buffer-injected larvae (data not shown). The DA neurons are present in locus caeruleus, olfactory bulb (Figure 6A, arrow), and pre-tectum in the telencephalon (Figure 6A and 6C-6D, arrowhead).

In contrast, the tyrosine hydroxylase-labeled olfactory bulb and pre-tectum was notably reduced and disappeared in *ddc* morphants (Figure 6B), and it could be rescued by co-injecting *ddc* mRNA (Figure 6D). In additional to the loss of tyrosine hydroxylase neurons in olfactory bulb and pre-tectum, the patterns of other DA neuron clusters were also altered in the ventral diencephalon. The DA neurons in the ventral diencephalon (dopaminergic neuron cluster 2-6) were regularly positioned around the midline. In contrast, there was abnormal patterning in *ddc* morphants and this patterning defect was partially restored by *ddc* mRNA co-injection (Figure 6D).

### Loss of Ddc causes seizure in zebrafish

**Figure 7:**
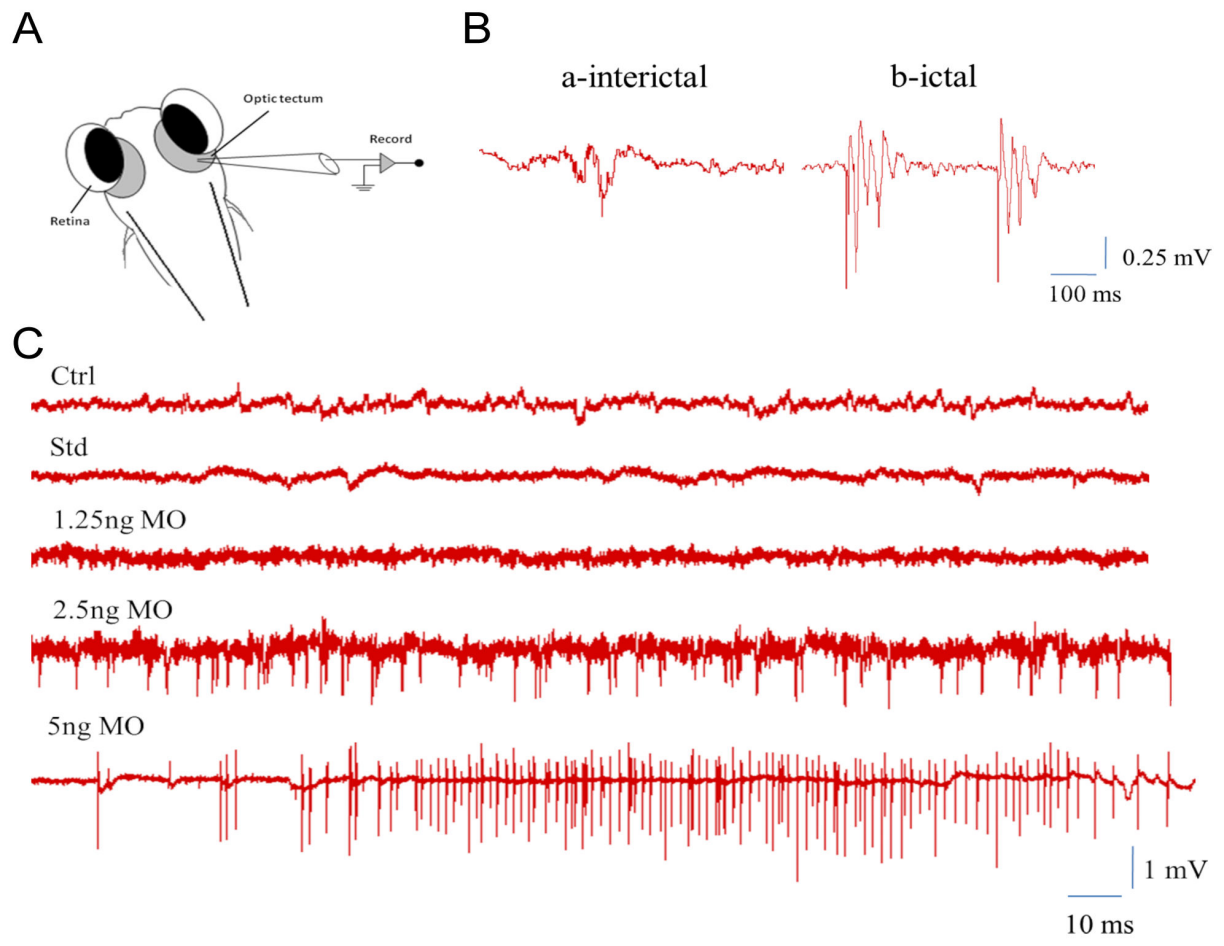
Epileptiform-like electrographic activity in zebrafish larvae. (**A**) Schematic representation of the conformation used to obtain optic tectum recordings from embedded larvae with 1% low melting agarose. (**B**) There were two different bursts which can be recorded: a-interictal for weak potential, and b-ictal for strong potential. (**C**) Recording to trace epileptiform-like electrographic activity by five different treatments, including control, standard MO, and 1.25, 2.5 and 5 ng *ddc* tMO1.

To examine if Ddc deficiency may also cause seizure activity in zebrafish, as reported in AADC patients [10], electrographic discharges at the optic tectum were recorded according to Manegold et al. [5]. The 7-dpf larvae showed two types of electrographic discharge, including a long-duration “ictal-like” and high-frequency, short-duration “inter-ictal-like” electrographic discharges, with low frequency and amplitude. In contrast, *ddc* tMO1 dose-dependently increased firing frequency and amplitude that resulted in seizure-like activities. At a higher dose (5 ng), the b-ictal response was the dominant form of discharge (Figure 7).

### Loss of Ddc impairs swimming activity

**Figure 8:**
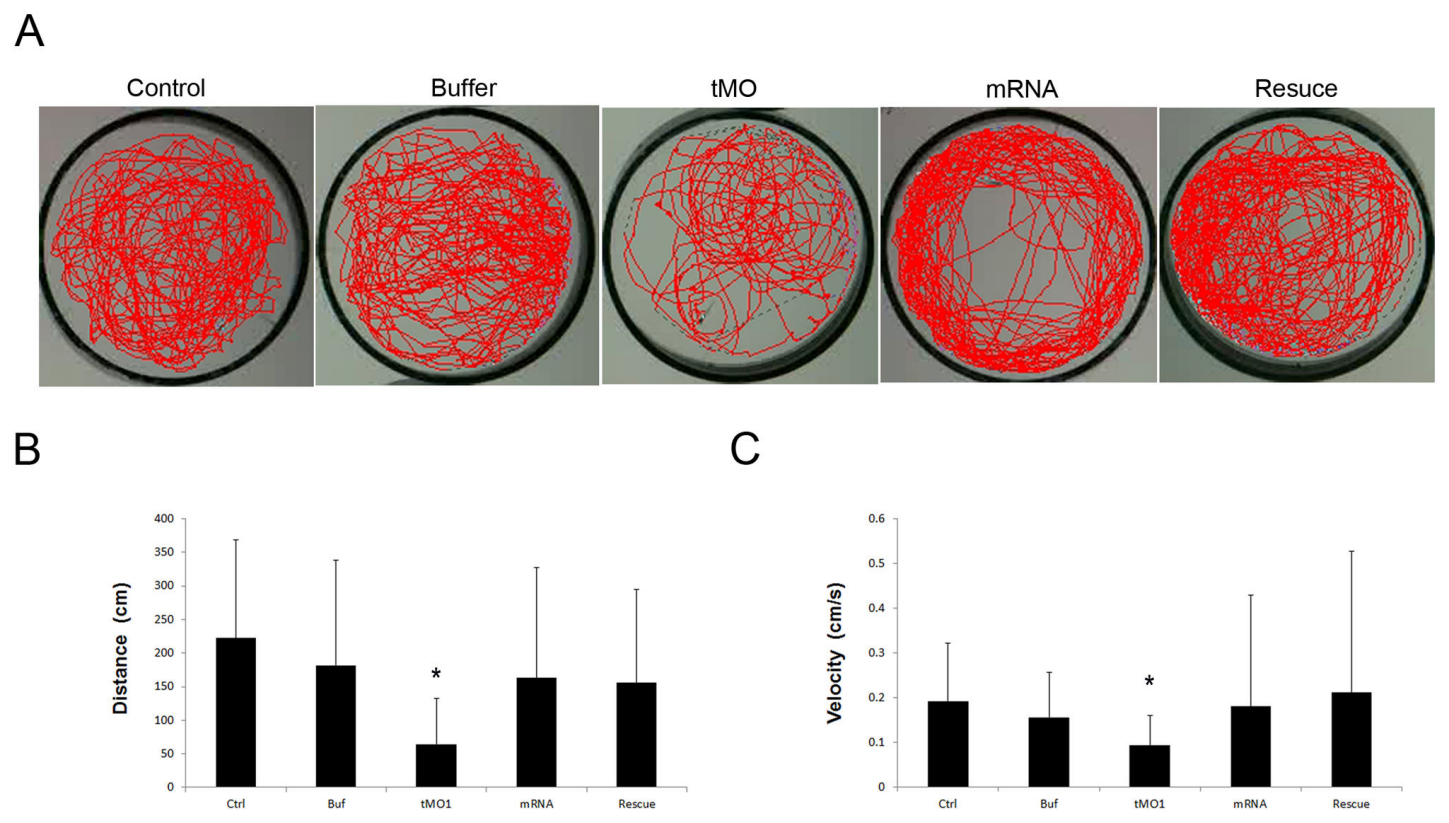
Behavioral evaluations in 5 dpf morphants showing the reduction of swimming ability. Both total distance and average velocity in swimming behavior were decreased in *ddc* morphants. (**A**) Swimming routes of a 5 dpf larva with injection of none, 1x injection buffer, 5 ng tMO1,100 pg mRNA and 5ng tMO1 and 100 pg *ddc* mRNA mixture to rescue, respectively. The video was recorded for 25 min. (**B**) Statistical analysis showed total distance of trajectories. The *ddc* morphants revealed decreased total distance of swimming compared with different controls. However, co-injection of 100 pg full length *ddc* mRNA can significantly attenuated the reduction of total swimming distance. (**C**) Statistical analysis for swimming velocity among different controls and *ddc* morphants, and rescued larvae also revealed the same results. The bars represent changes in different treatment of 5 dpf larvae in three independent experiments. n=31 for each treated larvae. Results were expressed as mean±SD. *p<0.05 compared to control larvae.

To further analyze the effect of Ddc knockdown on motor activity, the swimming behavior of 5-dpf zebrafish larvae were videotaped and analyzed using the Noldus Ethovision 3.1 software. Control and buffer-injected larvae swam actively as revealed by their respective swimming track (Figure 8A, n=31). In contrast, *ddc* tMO1 dose-dependently reduced their swimming activity. Both total swimming distance and velocity were reduced by the application of *ddc* tMO1 in a dose-dependent manner. This swimming defect was rescued by the co-injection of *ddc* mRNA (Figure 8B and 8C).

### Loss of DDC decreases eye movement

**Figure 9:**
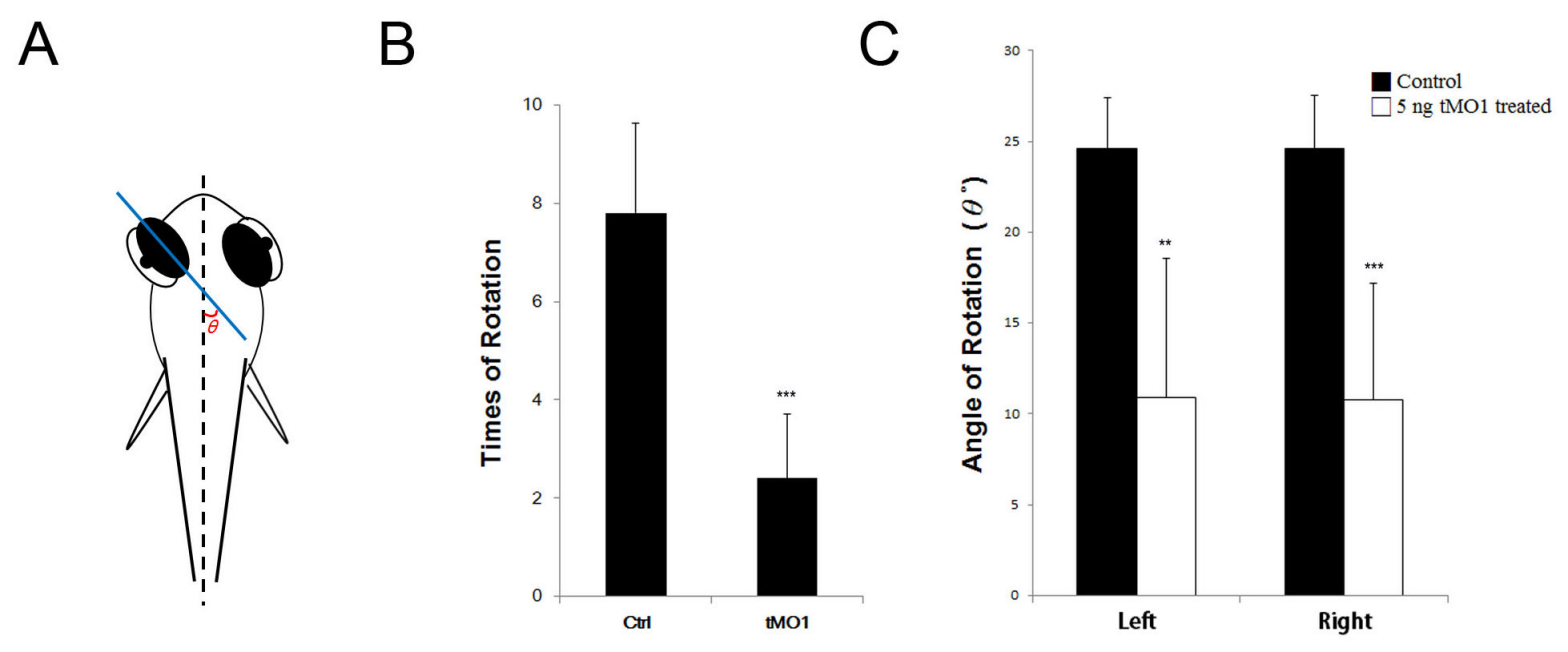
Eye movement in 5 dpf morphant significant reduced during rotation. Both frequency and angle of rotation decline compared with control and morpahant. (A) Schematic representation of the method used to obtain eye movement recordings from embedded larvae with 3% methylcellulose. Calculate with the angle θ to plane of eyeball and body axis. (B) Statistics analysis for eye rotation frequency during 120 seconds. (C) Statistics anaylsis for average of eye movement angle. Left: left eyeball, Right: right eyeball. The bars represent changes in different treatment of 5 dpf larvae in three independent experiments. n=7 for each treated larvae. Results were expressed as mean±SD. **p<0.01, ***p<0.001 compared to control larvae.

To investigate the effect of *ddc* MO on eye movement, 5-dpf zebrafish larvae were videotaped and analyzed by using the Tracker software. We measured 7 larvae in each group and found that the times of eye rotation and the rotating angle (*θ*) between to the middle plane of eyeball and fish midline as shown in Figure 9A were significantly reduced in morphants (Supplementary Movie S4) compared to that of control larvae (Supplementary Movie S3) (Figure 9B,C).

## Discussion

The pathogenetic studies of rare childhood diseases like AADC deficiency are often hindered by the lack of suitable animal models. Using pharmaceutical and anti-sense gene knockdown approaches, inhibiting AADC enzyme activity or gene expression results in defects in brain development, motor activity, and neuro-physiologic change in zebrafish, mimicking those observed in AADC deficiency patients [1,3,5,6,8–10,48]. It suggests that zebrafish could be a suitable model for studying AADC deficiency, especially for early neuronal development.

Several reports have shown that lacking biogenic monoamines like dopamine and serotonin-induced growth retardation in knockout mice [49–52]. The same phenomenon is noted in the present study. Furthermore, dopamine and serotonin also play important roles in brain growth during early stage of development [53–57]. Therefore, deficiency of dopamine and serotonin may lead to abnormal brain development.

Brain size had been reported to be increased by dietary dopamine and other substances supplement [58]. Patients with other neurologic diseases like attention-deficit and hyper-activity disorder, which reveal insufficient dopaminergic function, had been found to exhibit smaller brain size [59]. In AADC deficiency, 24% and 6% of children have abnormal brain as examined by magnetic resonance imaging (MRI) and computed tomography (CT scan), respectively [4,7].

Morphologic differences in the MRI in bilateral frontal lobes of some AADC-deficient children have also been reported [6]. Significant brain atrophy in the children can thus be attributed to a reduction in both dopamine and serotonin. In addition, AADC deficiency patients have higher L-Dopa and imbalanced glucose metabolism, which may also influence brain growth and functions [60]. Despite these clinical observations, no morphological or histo-pathological studies in early brain development in animal models have been done for the reduction of brain volume in AADC deficiency [49–52].

The current study demonstrates decreased total neuronal number and brain to white matter ratio, as well as increased apoptosis in *ddc* morphants compared to normal control. It indicates that reduced brain volume in *ddc* morphants may be related to increased apoptosis and impaired neurogenesis in zebrafish during the developmental stage. Serotonin has been shown to protect natural-killer cells by inhibiting apoptosis during neurogenesis [61]. The neurotoxicity of L-Dopa also increases apoptosis in neurons [62]. All of these factors may contribute to increased apoptosis in AADC deficiency.

Three sequential stereotype behaviors - spontaneous coiling contractions, touch-evoked response, and organized swimming - occur during zebrafish development [63]. Spontaneous contraction is entirely a reflex reaction and two other behaviors require hindbrain inputs to be elicited [64,65]. In this study, the *ddc* morphants have normal and even slightly hyperactive spontaneously coiling contractions (data not shown), which is consistent with clinical findings that most of AADC deficiency patients have normal, even hyper-reflex, response to stimulation [1,3,6,8].

To evaluate the effect of AADC inhibition on motor response of zebrafish, the area near head was touched to evoke an escape response in 3-dpf larvae [63]. The escape response to tactile stimulus warrants Mauthner cells, which are located in the rhombomere 4 of the hindbrain and are used to control the connection between the hindbrain and the spinal cord for escape reflex [64], and Rohon-Beard (R–B) sensory neurons, which are localized in the spinal cord [47]. The tyrosine hydroxylase and 5-hydroxytryptamine reactivity have been shown on Mauthner cells cluster and its serial homologue clusters MiD2cm and MiD3cm. Both enzymes’ reactivity have also been found in all levels of the sensorimotor pathway, including reticulo-spinal cells in the spinal cord, suggesting synaptic or non-synaptic effects of the aminergic system on motor neurons [47]. Thus, the decline in touch response may be due to the defects of connections between the hindbrain and spinal cord in Ddc-deficient zebrafish secondary to dopamine and serotonin deficiency.

Zebrafish larvae swim freely to change directions spontaneously starting from 27 hpf [63]. They develop beat-and-glide swimming toward their targets after 4 dpf [66] and then increase swimming frequency and feeding at 5 dpf [64]. Although dopamine can suppress swimming activity by activating D2 receptor at 3 dpf, the endogenous release of dopamine can modulate spontaneous swimming episodes at 5 dpf [67]. Impairment of dopaminergic neuronal development can thus result in swimming behavioral reduction [68].

The developmental appearance of another biogenic amine, serotonin, can also control locomotion. Serotonin can increase swimming episode frequency and modulate chloride homeostasis in zebrafish [69,70]. In contrast, 5-hydroxytryptamine antagonists methysergide and ketanserin can cause a decrease in the number of swimming occurrences [69]. Taken together, our results along with others suggest that the motility defects in *ddc* morphants may arise from the impairment of dopaminergic and serotoninergic neurons, consistent with the clinical findings in AADC patients [1,2,4,48]. Thus, the locomotion phenotypes of *ddc* morphants are analogous with the clinical AADC symptoms [5], suggesting that zebrafish is a useful model to illustrate the neuro-behavioral aspects of AADC deficiency and provide insights to the disease neuropathology.

Here, we observed DA neuronal patterning defects in *ddc* morphants. The loss of locomotive abilities may also be partly linked to the progressive abnormality of DA neuron development [68]. The DA neurons can be grouped into two families. The first group of DA neurons is the D1-like family expressing D1 and D5 receptors [71]. The second group is the D2-like family expressing D2, D3 and D4 receptors, which have been shown to regulate locomotion [72,73]. Some DA neuron clusters have decreased or lost expression of tyrosine hydroxylase protein in Ddc-deficient larvae, including the olfactory bulb and pre-tectum and DA neuron clusters 1 in the ventral diencephalon, which expresses D2 and D3 receptors [73,74]. The other possible explanations may be that reduction in AADC levels in zebrafish also leads to a selective loss of tyrosine hydroxylase protein expression. Taken together, these findings suggest that dopaminergic structural changes resulting from AADC deficiency may also contribute to the pathogenetic mechanisms of neurologic manifestations in AADC deficiency. However, neuronal pattering is changing during development, we cannot exclude the possibility these observed changes might be affected by the growth retardation of *ddc* morphants.

Seizure-like activity is also demonstrated in *ddc* morphants. Although epileptic seizures are uncommon in AADC deficiency [4,10], patients have frequent myoclonic movements resembling seizure activity, which may be misdiagnosed as seizures during the early stages. Seizure-like activity suggests that AADC deficiency may increase excitatory activity in neurons, as seen in patients with AADC deficiency. In addition, it might also influence the abnormal swimming behaviors observed in *ddc* morphants.

During development, the visual behavior is nearly matured after 79 hpf with characteristic twisting of eyeballs every 10 sec and 20-30 degree eye rotation every 20 sec [75,76]. Here, we observed significantly reduced eye movements in *ddc* marphants. Episodes of oculogyric crises were major symptom in many AADC deficiency patients [4–6]. It further supports the use of zebrafish as a model for AADC deficiency.

In summary, changes in motor behavior and brain structure of *ddc* morphants corroborate the complicated role of AADC in neuronal development. This model expresses many aspects of clinical and functional manifestations in AADC deficiency. The morphologic and behavioral features of zebrafish may provide valuable insights for assessing early neuronal development.

## Materials and Methods

### Ethics Statement

All animal handling procedures were approved by the use of laboratory animal committee at National Taiwan University, Taipei, Taiwan (IACUC Approval ID: 100 Animal Use document No. 53).

### Fish strains and maintenance

The AB cross TU strain of zebrafish were used for all experiments and kept at 26-28°C. Embryos were collected by natural spawning and raised according to standard procedures [77]. They were staged in hours or days post fertilization according to standard criteria (Kimmel et al., 1995). Embryos and larvae were cultured at 28.5° C.

### Isolation and sequence analysis

The sequence of human AADC protein (accession number BC008366.1) was blasted to the zebrafish genome using the Ensembl database (http://www.ensembl.org/Danio_rerio/blastview). A conceptual translation product of a single annotated transcript was identified as the top match. The predicted zebrafish protein sequence was aligned with *human, mice*, *Drosophila* and *Xenopus* AADC protein sequences using the ClustalW2 software (www.clustal.org). The chromosomal location of the predicted zebrafish *ddc* transcript was aligned with the zebrafish genome to determine its exon-intron boundaries and splice sites.

### RT-PCR analysis

Total RNAs were prepared from adult tissues and early embryos using the TRIzol reagent (Invitrogen Corporation, Carlsbad, CA). To synthesize single-strand cDNAs, 3 µg of total RNAs, oligo dT primers and M-MLV Reverse Transcriptase (Promega Corporation, Madison, WI) were applied in a total reaction volume of 25 µL. The presence of *ddc* transcripts in different cDNA samples was detected by amplification using a zebrafish *ddc* primer pair (5’- ATAGAATTCTACTAAAAGATGGATGCCGC-3’ (forward) and 5’- ATATCTAGATCCGTGTAGCAGTTCCTGAA-3’ (reverse) by PCR.

A sequence analysis for 25 cycles at a thermal cycler (PTC-200, MJ Research) was manipulated according to the following protocol: denatured at 94° C for 30 sec, annealed at 55~60°C for 30 sec, and elongated at 72° C for 1 min with *ef1α* (524 bp, 5’-CAAGGAAGTCAGCGCA TACA-3’ and 5’-TGATGACCTGAG CGTTGAAG-3’) as an internal control. The PCR products were analyzed on 1.0% (w/v) agarose gels stained with ethidium bromide.

### Pharmacologic treatment to inhibit AADC activity

The AADC inhibitor, NSD-1015 (Sigma, USA) was dissolved as 1 mM stock in 0.3 X Danieaus’ buffer. After dilution by Danieaus’ buffer from 500 to 100 µM, dechorionated larvae were treated at 10 hpf. The inhibitor solution at 24 hpf was changed every 24 hours to maintain the effect of the compound.

### MO-mediated knockdown of ddc expression

Morpholino, anti-sense oligonucleotides, (MO, GeneTools LLC, Philomath, OR, USA) were designed by GeneTools to block translation of zebrafish *ddc* by targeting at ATG site. The MO sequences were: 5’–3’ (Table S1). Standard MOs with five mismatches were used in order to distinguish the phenotypic effects specific to the knockdown of Ddc from the effects of non-specific MO toxicity. The MOs were re-suspended in sterile water at 1mM stock concentration. Immediately prior to injection, tMO1 and tMO2 were diluted to serial concentration and saturated phenol red (Sigma, Poole, UK) with embryonic medium (13.7 mM NaCl, 0.54 mM KCl, 0.025 mM Na_2_HPO_4_, 0.044 mM KH_2_PO_4_, 1.3 mM CaCl_2,_ 1.0 mM MgSO_4_ and 0.42 mM NaHCO_3_) were added to monitor injection efficiency. Full-length coding sequences of *ddc* with 5’-UTR MO binding regions were cloned from zebrafish cDNAs into the pCS2^+^ -GFP vector for MO efficiency check (Figure S1). The MOs were injected into the yolk of one- to four-cell stage embryos. Both two *ddc* tMOs were injected at 0.5 mM final concentration.

### AADC activity assay

To test the effectiveness of AADC inhibitor NSD-1015, 3-dpf zebrafish embryos were manually dechorionated, fractured by repeated suction via a 200 µL micropipette tip in phosphate-buffered saline (PBS), washed twice by centrifugation and resuspended in PBS. The AADC activity assay was modified from a previous study [9]. Briefly, embryo lysates were then centrifuged again and resuspended in 0.32 M sucrose, sonicated, and mixed with 10 mM L-Dopa and 0.7 mM pyridoxal 5-phosphate at 37° C for 2 h with or without NSD-1015. The treated lysates were subjected to high performance liquid chromatography (HPLC) (bioanalytical systems, Inc) and the dopamine peaks were detected using eletrochemical detection by PM-80 solvent delivery system and subjected to Clarity LITE software for analysis.

To measure the AADC activity in *ddc* MO-injected embryos, 1-cell stage zebrafish embryos were microinjected with designated amount of *ddc* tMO1, cultured to 3 dpf and subjected to AADC activity assay as previously described.

### Electrophysiology

To obtain stable physiologic recordings, zebrafish larvae (7 dpf) were immobilized in 1% low melting temperature agarose. Zebrafish larvae were placed in agarose and were accessible for electrode placement. Fishes immobilized in agar maintained in normal bathing medium remained viable for ≤24 h. Under direct visual guidance, a glass microelectrode (~1-µm tip diameter, 2-7 Ω) was placed in the optic tectum, the largest midbrain structure in the zebrafish central nervous system. Electrodes were filled with 2 M NaCl and electrical activities were recorded for 30 min in each experiment.

### ddc mRNA for rescue

The full length of *ddc* with 5 point mutations that does not bind with anti-sense MO was cloned into the pcDNA construct. The capped mRNA were transcribed by T7 polymerase (mMESSAGE mMACHINE™, Ambion) and dissolved in nucleotide-free water. Embryos were injected with capped mRNA at one-cell stage and collected at different developmental stages for rescue experiments.

### Whole- mount *in situ* hybridization

The embryos were staged by morphology [78] and fixed in 4% paraformaldehyde in phosphate-buffered saline (PBS) at 4° C overnight, then transferred to 100% methanol for storage at -20° C for at least 24 h before undergoing hybridization.

Whole-mount *in situ* hybridization of zebrafish embryos was performed as described previously [79]. A digoxigenin-labeled anti-sense RNA probe was synthesized from the *ddc* plasmid, linearized by XhoI, and transcribed using SP6 RNA polymerase (Invitrogen, Paisley, UK). The embryos were mounted in glycerol and photographed by the Canon 405 system.

### Evaluation of locomotor behavior

Touch response and swimming behavior were investigated in NSD-1015-treated embryos and *ddc* morphants (*ddc* MO-injected embryos) at 3dpf and 5 dpf, respectively, in embryonic medium. The trigeminal nerve was touched behind the fish eyes using the needle tip to perform touch response. The treated larvae were put in a translucent plastic dish filled with 4 ml Danieaus’ buffer and placed on a light stage with x-ray light box. Larvae swam in a 3 cm-wide circular arena, were allowed to acclimate in the dish for 5 min, and swimming behavior was then recorded for 25 min using an iphone 3GS camera.

File analysis by Ethovision Pro 4.01 digital video recording software were used to track movement (Tracksys, Nottingham, UK). All digital tracks were analyzed for total distances moved using an input filter of 0.06 cm as the minimum distance to be considered “movement”. Each experiment was performed in triplicate and the results were expressed as mean total movements±SEM.

### Analysis for eye movement

5-dpf.control and *ddc* morphant larvae were fixed in 3% methylcellulose, videotaped using Canon 405 system and analyzed by the Tracker 4.80 (http://www.cabrillo.edu/~dbrown/tracker/). The number of eye twisting and the twisting angle of eyes were measured by an angle between the middle plane of the eyeball and the fish body midline.

### Plastic section for brain structure analysis

Zebrafish larvae were fixed in 4% paraformaldehyde/ PBS and then dehydrated with 100% methanol at -20° C for at least 24 h. After dehydration, the larvae were embedded in Technovit 7100 resin kit at dark (Heraeus Kulzer). The larvae were sectioned from the most anterior larvae head to the spinal cord by 5 µm using the Microm HM360 (Thermo science, USA) and stained with toluidine blue at intervals. The brain to white matter ratio of zebrafish larvae was measured using plastic sections containing chiasma. The areas of brain and white matter were measured using the ImageJ software and their ratios were calculated.

### Apoptosis assay

The larvae were fixed in fresh 4% paraformaldehyde in PBS-T. After treatment with 10 µg/ml protease for 5 min, the embryos were post-fixed by postfix solution (acetic acid: ethanol 1:2) for 10 min at -20 °C. They were then washed in PBS and subjected to TUNEL assay using the *in situ* cell death detection kit (Roche) according manufacturer’s instructions.

### Quantification and statistical analysis

All data were analyzed using the ImageJ Software (NIH) and calculated for each larva. The SPS9.2 system was used for data quantification and statistical analysis. The statistical analysis was done using one-way ANOVA and Student’s t-test. Data were expressed as mean±SD and all experiments were independently repeated at least three times. Statistical significance was set at *p*<0.05.

## Supporting Information

Table S1 **Sequences of morpholino oligonucleotides** and **anti-sense probes for whole-mount in situ hybridization analysis used in this study.**
(PDF)

Figure S1 **Efficiency testing of *ddc* translational blocking MOs.** (A) A 303 bp partial sequence of *ddc* containing tMO1 or tMO2 targeting site (purple region) was inserted into a pCS2^+^ vector containing CMV promoter (brown region) and GFP (green region) sequences. The binding sequences for *ddc* tMO1 and tMO2 are shown below the construct map. (B) Embryos were co-injected with designated MO (5 ng) and MO efficiency testing plasmid (100 pg), examined by epifluorescent microscope under bright and dark fields. Representative superimposed images are shown. (C) Percentages of embryos showing GFP in different treatments are shown. Plasmid injected only group, n=106; tMO1,n=103; tMO2, N=64. * *p* <0.001.
(PDF)

Figure S2 ***ddc* MO caused reduction in brain size as examined by whole-mount *in situ* hybridization against *huc*.** Embryos were treated as indicated and subjected to whole-mount is situ hybridization against *huc*.
(PDF)

Movie S1 **A control zebrafish larva (3 days old) was poked on the trigeminal neuron posterior to the eyes and a rapid escaping response was observed in a 30-sec recording.**
(AVI)

Movie S2 **A NSD-1015 treated zebrafish larva (3 days old) was poked on the trigeminal neuron posterior to the eyes and no escaping response was observed in a 30-sec recording.**
(AVI)

Movie S3 **A control zebrafish larva (5 days old) was native twist the eyes were observed in a 120-sec recording.**
(AVI)

Movie S4 **A 5ng tMO1 treated zebrafish larva (5 days old) was native twist the eyes were observed in a 120-sec recording.**
(AVI)

## References

[1] Pons R, Ford B, Chiriboga CA, Clayton PT, Hinton V et al. (2004) Aromatic L-amino acid decarboxylase deficiency: clinical features, treatment, and prognosis. Neurology 62: 1058-1065. doi:10.1212/WNL.62.7.1058. PubMed: 15079002. 15079002 10.1212/wnl.62.7.1058

[2] Swoboda KJ, Hyland K, Goldstein DS, Kuban KC, Arnold LA et al. (1999) Clinical and therapeutic observations in aromatic L-amino acid decarboxylase deficiency. Neurology 53: 1205-1211. doi:10.1212/WNL.53.6.1205. PubMed: 10522874. 10522874 10.1212/wnl.53.6.1205

[3] Swoboda KJ, Saul JP, McKenna CE, Speller NB, Hyland K (2003) Aromatic L-amino acid decarboxylase deficiency: overview of clinical features and outcomes. Ann Neurol 54 Suppl 6: S49-S55. doi:10.1002/ana.10631. PubMed: 12891654. 12891654 10.1002/ana.10631

[4] Brun L, Ngu LH, Keng WT, Ch'ng GS, Choy YS, et al. (2010) Clinical and biochemical features of aromatic L-amino acid decarboxylase deficiency. Neurology 75: 64-71. doi:10.1212/WNL.0b013e3181e620ae. PubMed: 20505134. 20505134 10.1212/WNL.0b013e3181e620ae

[5] Manegold C, Hoffmann GF, Degen I, Ikonomidou H, Knust A et al. (2009) Aromatic L-amino acid decarboxylase deficiency: clinical features, drug therapy and follow-up. J Inherit Metab Dis 32: 371-380. doi:10.1007/s10545-009-1076-1. PubMed: 19172410. 19172410 10.1007/s10545-009-1076-1

[6] Lee HF, Tsai CR, Chi CS, Chang TM, Lee HJ (2009) Aromatic L-amino acid decarboxylase deficiency in Taiwan. Eur J Paediatr Neurol 13: 135-140. doi:10.1016/j.ejpn.2008.03.008. PubMed: 18567514. 18567514 10.1016/j.ejpn.2008.03.008

[7] Lee WT, Weng WC, Peng SF, Tzen KY (2009) Neuroimaging findings in children with paediatric neurotransmitter diseases. J Inherit Metab Dis 32: 361-370. doi:10.1007/s10545-009-1106-z. PubMed: 19455403. 19455403 10.1007/s10545-009-1106-z

[8] Hyland K, Clayton PT (1990) Aromatic amino acid decarboxylase deficiency in twins. J Inherit Metab Dis 13: 301-304. doi:10.1007/BF01799380. PubMed: 1700191. 1700191 10.1007/BF01799380

[9] Verbeek MM, Geurtz PB, Willemsen MA, Wevers RA (2007) Aromatic L-amino acid decarboxylase enzyme activity in deficient patients and heterozygotes. Mol Genet Metab 90: 363-369. doi:10.1016/j.ymgme.2006.12.001. PubMed: 17240182. 17240182 10.1016/j.ymgme.2006.12.001

[10] Ito S, Nakayama T, Ide S, Ito Y, Oguni H et al. (2008) Aromatic L-amino acid decarboxylase deficiency associated with epilepsy mimicking non-epileptic involuntary movements. Dev Med Child Neurol 50: 876-878. doi:10.1111/j.1469-8749.2008.03094.x. PubMed: 18754761. 18754761 10.1111/j.1469-8749.2008.03094.x

[11] Crisp KM, Mesce KA (2004) A cephalic projection neuron involved in locomotion is dye coupled to the dopaminergic neural network in the medicinal leech. J Exp Biol 207: 4535-4542. doi:10.1242/jeb.01315. PubMed: 15579549. 15579549 10.1242/jeb.01315

[12] Kiehn O, Kjaerulff O (1996) Spatiotemporal characteristics of 5-HT and dopamine-induced rhythmic hindlimb activity in the in vitro neonatal rat. J Neurophysiol 75: 1472-1482. PubMed: 8727391. 8727391 10.1152/jn.1996.75.4.1472

[13] Marder E, Eisen JS (1984) Electrically coupled pacemaker neurons respond differently to same physiological inputs and neurotransmitters. J Neurophysiol 51: 1362-1374. PubMed: 6145758. 6145758 10.1152/jn.1984.51.6.1362

[14] Schotland J, Shupliakov O, Wikström M, Brodin L, Srinivasan M et al. (1995) Control of lamprey locomotor neurons by colocalized monoamine transmitters. Nature 374: 266-268. doi:10.1038/374266a0. PubMed: 7885446. 7885446 10.1038/374266a0

[15] Verney C, Lebrand C, Gaspar P (2002) Changing distribution of monoaminergic markers in the developing human cerebral cortex with special emphasis on the serotonin transporter. Anat Rec 267: 87-93. doi:10.1002/ar.10089. PubMed: 11997877. 11997877 10.1002/ar.10089

[16] Gaspar P, Cases O, Maroteaux L (2003) The developmental role of serotonin: news from mouse molecular genetics. Nat Rev Neurosci 4: 1002-1012. doi:10.1038/nrn1256. PubMed: 14618156. 14618156 10.1038/nrn1256

[17] Neff NH, Hadjiconstantinou M (1995) Aromatic L-amino acid decarboxylase modulation and Parkinson’s disease. Prog Brain Res 106: 91-97. doi:10.1016/S0079-6123(08)61206-6. PubMed: 8584678. 8584678 10.1016/s0079-6123(08)61206-6

[18] King JM, Muthian G, Mackey V, Smith M, Charlton C (2011) L-Dihydroxyphenylalanine modulates the steady-state expression of mouse striatal tyrosine hydroxylase, aromatic L-amino acid decarboxylase, dopamine and its metabolites in an MPTP mouse model of Parkinson’s disease. Life Sci 89: 638-643. doi:10.1016/j.lfs.2011.08.008. PubMed: 21871902. 21871902 10.1016/j.lfs.2011.08.008 PMC3189304

[19] Kitahama K, Ikemoto K, Jouvet A, Araneda S, Nagatsu I et al. (2009) Aromatic L-amino acid decarboxylase-immunoreactive structures in human midbrain, pons, and medulla. J Chem Neuroanat 38: 130-140. doi:10.1016/j.jchemneu.2009.06.010. PubMed: 19589383. 19589383 10.1016/j.jchemneu.2009.06.010

[20] Moreira-Rodrigues M, Sampaio-Maia B, Pestana M (2009) Renal dopaminergic system activity in rat remnant kidney up to twenty-six weeks after surgery. Life Sci 84: 409-414. doi:10.1016/j.lfs.2008.12.018. PubMed: 19167406. 19167406 10.1016/j.lfs.2008.12.018

[21] Yang S, Yao B, Zhou Y, Yin H, Zhang MZ et al. (2012) Intrarenal dopamine modulates progressive angiotensin II-mediated renal injury. Am J Physiol Renal Physiol 302: F742-F749. doi:10.1152/ajprenal.00583.2011. PubMed: 22169008. 22169008 10.1152/ajprenal.00583.2011 PMC3311314

[22] Blechingberg J, Holm IE, Johansen MG, Børglum AD, Nielsen AL (2010) Aromatic l-amino acid decarboxylase expression profiling and isoform detection in the developing porcine brain. Brain Res 1308: 1-13. doi:10.1016/j.brainres.2009.10.051. PubMed: 19857468. 19857468 10.1016/j.brainres.2009.10.051

[23] Eberling JL, Roberts JA, Taylor SE, VanBrocklin HF, O’Neil JP et al. (2002) No effect of age and estrogen on aromatic L- amino acid decarboxylase activity in rhesus monkey brain. Neurobiol Aging 23: 479-483. doi:10.1016/S0197-4580(01)00323-2. PubMed: 11959410. 11959410 10.1016/s0197-4580(01)00323-2

[24] Waymire JC, Gilmer-Waymire K (1978) Adrenergic enzymes in cultured mouse neuroblastoma: absence of detectable aromatic-L-amino-acid decarboxylase. J Neurochem 31: 693-698. doi:10.1111/j.1471-4159.1978.tb07842.x. PubMed: 28384. 28384 10.1111/j.1471-4159.1978.tb07842.x

[25] Chatelin S, Wehrlé R, Mercier P, Morello D, Sotelo C et al. (2001) Neuronal promoter of human aromatic L-amino acid decarboxylase gene directs transgene expression to the adult floor plate and aminergic nuclei induced by the isthmus. Brain Res Molecular Brain Res 97: 149-160. doi:10.1016/S0169-328X(01)00318-7. PubMed: 11750071. 10.1016/s0169-328x(01)00318-7 11750071

[26] Takezako T, Noda K, Tsuji E, Koga M, Sasaguri M et al. (2001) Adenosine activates aromatic L-amino acid decarboxylase activity in the kidney and increases dopamine. J Am Soc Nephrol 12: 29-36. PubMed: 11134247. 11134247 10.1681/ASN.V12129

[27] Costa M, Furness JB, McLean JR (1976) The presence of aromatic L-amino acid decarboxylase in certain intestinal nerve cells. Histochemistry 48: 129-143. doi:10.1007/BF00494551. PubMed: 955982. 955982 10.1007/BF00494551

[28] Hayashi H, Mizuguchi H, Kagamiyama H (1993) Rat liver aromatic L-amino acid decarboxylase: spectroscopic and kinetic analysis of the coenzyme and reaction intermediates. Biochemistry 32: 812-818. doi:10.1021/bi00054a011. PubMed: 8422386. 8422386 10.1021/bi00054a011

[29] Gilbert JA, Frederick LM, Ames MM (2000) The aromatic-L-amino acid decarboxylase inhibitor carbidopa is selectively cytotoxic to human pulmonary carcinoid and small cell lung carcinoma cells. Clin Cancer Res Off J Am Assoc Cancer Res 6: 4365-4372. PubMed: 11106255. 11106255

[30] Eberle ED, Eiduson S (1968) Effect of pyridoxine deficiency on aromatic L-amino acid decarboxylase in the developing rat liver and brain. J Neurochem 15: 1071-1083. doi:10.1111/j.1471-4159.1968.tb06825.x. PubMed: 5711122. 5711122 10.1111/j.1471-4159.1968.tb06825.x

[31] DeJesus OT, Flores LG, Roberts AD, Dick DW, Bartlett RM et al. (2005) Aromatic L-amino acid decarboxylase (AAAD) activity in rhesus macaque striatum after MAO-B inhibition by Ro 16-6491. Synapse 56: 54-56. doi:10.1002/syn.20119. PubMed: 15700282. 15700282 10.1002/syn.20119

[32] Eaton MJ, Gudehithlu KP, Quach T, Silvia CP, Hadjiconstantinou M et al. (1993) Distribution of aromatic L-amino acid decarboxylase mRNA in mouse brain by in situ hybridization histology. J Comp Neurol 337: 640-654. doi:10.1002/cne.903370409. PubMed: 7904615. 7904615 10.1002/cne.903370409

[33] Cañestro C, Yokoi H, Postlethwait JH (2007) Evolutionary developmental biology and genomics. Nat Rev Genet 8: 932-942. doi:10.1038/nrg2226. PubMed: 18007650. 18007650 10.1038/nrg2226

[34] Whitlock KE (2004) Development of the nervus terminalis: origin and migration. Microsc Res Tech 65: 2-12. doi:10.1002/jemt.20094. PubMed: 15570589. 15570589 10.1002/jemt.20094

[35] Rinkwitz S, Mourrain P, Becker TS (2011) Zebrafish: an integrative system for neurogenomics and neurosciences. Prog Neurobiol 93: 231-243. doi:10.1016/j.pneurobio.2010.11.003. PubMed: 21130139. 21130139 10.1016/j.pneurobio.2010.11.003

[36] Barinaga M (1990) Zebrafish: swimming into the development mainstream. Science 250: 34-35. doi:10.1126/science.2218513. PubMed: 2218513. 2218513 10.1126/science.2218513

[37] Müller UK, van Leeuwen JL (2004) Swimming of larval zebrafish: ontogeny of body waves and implications for locomotory development. J Exp Biol 207: 853-868. doi:10.1242/jeb.00821. PubMed: 14747416. 14747416 10.1242/jeb.00821

[38] Yamamoto K, Ruuskanen JO, Wullimann MF, Vernier P (2010) Two tyrosine hydroxylase genes in vertebrates New dopaminergic territories revealed in the zebrafish brain. Mol Cell Neurosci 43: 394-402. doi:10.1016/j.mcn.2010.01.006. PubMed: 20123022. 20123022 10.1016/j.mcn.2010.01.006

[39] Yamamoto K, Ruuskanen JO, Wullimann MF, Vernier P (2011) Differential expression of dopaminergic cell markers in the adult zebrafish forebrain. J Comp Neurol 519: 576-598. doi:10.1002/cne.22535. PubMed: 21192085. 21192085 10.1002/cne.22535

[40] Schweitzer J, Driever W (2009) Development of the dopamine systems in zebrafish. Adv Exp Med Biol 651: 1-14. doi:10.1007/978-1-4419-0322-8_1. PubMed: 19731546. 19731546 10.1007/978-1-4419-0322-8_1

[41] Rink E, Wullimann MF (2001) The teleostean (zebrafish) dopaminergic system ascending to the subpallium (striatum) is located in the basal diencephalon (posterior tuberculum). Brain Res 889: 316-330. doi:10.1016/S0006-8993(00)03174-7. PubMed: 11166725. 11166725 10.1016/s0006-8993(00)03174-7

[42] Schweitzer J, Lohr H, Filippi A, Driever W (2012) Dopaminergic and noradrenergic circuit development in zebrafish. Dev Neurobiol 72: 256-268. doi:10.1002/dneu.20911. PubMed: 21567980. 21567980 10.1002/dneu.20911

[43] Lin YL, Gao J (2010) Internal proton transfer in the external pyridoxal 5'-phosphate Schiff base in dopa decarboxylase. Biochemistry 49: 84-94. doi:10.1021/bi901790e. PubMed: 19938875. 19938875 10.1021/bi901790e PMC2827857

[44] Miñano FJ, McMillen BA, Myers RD (1990) Central action of an inhibitor of brain dopa-decarboxylase, NSD-1015, on cyanamide-induced alcohol drinking in rats. Pharmacol Biochem Behav 35: 465-468. doi:10.1016/0091-3057(90)90186-L. PubMed: 2320657. 2320657 10.1016/0091-3057(90)90186-l

[45] Hosseini-Sharifabad M, Nyengaard JR (2007) Design-based estimation of neuronal number and individual neuronal volume in the rat hippocampus. J Neurosci Methods 162: 206-214. doi:10.1016/j.jneumeth.2007.01.009. PubMed: 17368561. 17368561 10.1016/j.jneumeth.2007.01.009

[46] Chen Q, Huang NN, Huang JT, Chen S, Fan J et al. (2009) Sodium benzoate exposure downregulates the expression of tyrosine hydroxylase and dopamine transporter in dopaminergic neurons in developing zebrafish. Birth Defects Res B Dev Reprod Toxicol 86: 85-91. doi:10.1002/bdrb.20187. PubMed: 19294673. 19294673 10.1002/bdrb.20187

[47] McLean DL, Fetcho JR (2004) Relationship of tyrosine hydroxylase and serotonin immunoreactivity to sensorimotor circuitry in larval zebrafish. J Comp Neurol 480: 57-71. doi:10.1002/cne.20281. PubMed: 15514919. 15514919 10.1002/cne.20281

[48] Korenke GC, Christen HJ, Hyland K, Hunneman DH, Hanefeld F (1997) Aromatic L-amino acid decarboxylase deficiency: an extrapyramidal movement disorder with oculogyric crises. Eur J Paediatr Neurol 1: 67-71. doi:10.1016/S1090-3798(97)80065-7. PubMed: 10728198. 10728198

[49] Yang S, Lee YJ, Kim JM, Park S, Peris J et al. (2006) A murine model for human sepiapterin-reductase deficiency. Am J Hum Genet 78: 575-587. doi:10.1086/501372. PubMed: 16532389. 16532389 10.1086/501372 PMC1424682

[50] Alenina N, Kikic D, Todiras M, Mosienko V, Qadri F et al. (2009) Growth retardation and altered autonomic control in mice lacking brain serotonin. Proc Natl Acad Sci U S A 106: 10332-10337. doi:10.1073/pnas.0810793106. PubMed: 19520831. 19520831 10.1073/pnas.0810793106 PMC2700938

[51] Sumi-Ichinose C, Urano F, Shimomura A, Sato T, Ikemoto K et al. (2005) Genetically rescued tetrahydrobiopterin-depleted mice survive with hyperphenylalaninemia and region-specific monoaminergic abnormalities. J Neurochem 95: 703-714. doi:10.1111/j.1471-4159.2005.03402.x. PubMed: 16135092. 16135092 10.1111/j.1471-4159.2005.03402.x

[52] Kwak SS, Suk J, Choi JH, Yang S, Kim JW et al. (2011) Autophagy induction by tetrahydrobiopterin deficiency. Autophagy 7: 1323-1334. doi:10.4161/auto.7.11.16627. PubMed: 21795851. 21795851 10.4161/auto.7.11.16627 PMC3242797

[53] Airhart MJ, Lee DH, Wilson TD, Miller BE, Miller MN et al. (2012) Adverse effects of serotonin depletion in developing zebrafish. Neurotoxicol Teratol 34: 152-160. doi:10.1016/j.ntt.2011.08.008. PubMed: 21893190. 21893190 10.1016/j.ntt.2011.08.008

[54] Flinn L, Mortiboys H, Volkmann K, Köster RW, Ingham PW et al. (2009) Complex I deficiency and dopaminergic neuronal cell loss in parkin-deficient zebrafish (Danio rerio). Brain J Neurol 132: 1613-1623. doi:10.1093/brain/awp108. PubMed: 19439422. 10.1093/brain/awp108 19439422

[55] Russek-Blum N, Gutnick A, Nabel-Rosen H, Blechman J, Staudt N et al. (2008) Dopaminergic neuronal cluster size is determined during early forebrain patterning. Development 135: 3401-3413. doi:10.1242/dev.024232. PubMed: 18799544. 18799544 10.1242/dev.024232 PMC2692842

[56] Crandall JE, McCarthy DM, Araki KY, Sims JR, Ren JQ et al. (2007) Dopamine receptor activation modulates GABA neuron migration from the basal forebrain to the cerebral cortex. J Neurosci Off J Soc Neurosci 27: 3813-3822. doi:10.1523/JNEUROSCI.5124-06.2007. PubMed: 17409246. 10.1523/JNEUROSCI.5124-06.2007 PMC2711976 17409246

[57] Araki KY, Sims JR, Bhide PG (2007) Dopamine receptor mRNA and protein expression in the mouse corpus striatum and cerebral cortex during pre- and postnatal development. Brain Res 1156: 31-45. doi:10.1016/j.brainres.2007.04.043. PubMed: 17509542. 17509542 10.1016/j.brainres.2007.04.043 PMC1994791

[58] Previc FH (1999) Dopamine and the origins of human intelligence. Brain Cogn 41: 299-350. doi:10.1006/brcg.1999.1129. PubMed: 10585240. 10585240 10.1006/brcg.1999.1129

[59] Swanson JM, Kinsbourne M, Nigg J, Lanphear B, Stefanatos GA et al. (2007) Etiologic subtypes of attention-deficit/hyperactivity disorder: brain imaging, molecular genetic and environmental factors and the dopamine hypothesis. Neuropsychol Rev 17: 39-59. doi:10.1007/s11065-007-9019-9. PubMed: 17318414. 17318414 10.1007/s11065-007-9019-9

[60] Ide S, Sasaki M, Kato M, Shiihara T, Kinoshita S et al. (2010) Abnormal glucose metabolism in aromatic L-amino acid decarboxylase deficiency. Brain Dev 32: 506-510. doi:10.1016/j.braindev.2009.05.004. PubMed: 19520530. 19520530 10.1016/j.braindev.2009.05.004

[61] Betten A, Dahlgren C, Hermodsson S, Hellstrand K (2001) Serotonin protects NK cells against oxidatively induced functional inhibition and apoptosis. J Leukoc Biol 70: 65-72. PubMed: 11435487. 11435487

[62] Zhang M, Lee HJ, Park KH, Park HJ, Choi HS et al. (2012) Modulatory effects of sesamin on dopamine biosynthesis and L-DOPA-induced cytotoxicity in PC12 cells. Neuropharmacology 62: 2219-2226. doi:10.1016/j.neuropharm.2012.01.012. PubMed: 22293035. 22293035 10.1016/j.neuropharm.2012.01.012

[63] Saint-Amant L, Drapeau P (1998) Time course of the development of motor behaviors in the zebrafish embryo. J Neurobiol 37: 622-632. doi:10.1002/(SICI)1097-4695(199812)37:4. PubMed: 9858263. 9858263 10.1002/(sici)1097-4695(199812)37:4<622::aid-neu10>3.0.co;2-s

[64] Drapeau P, Saint-Amant L, Buss RR, Chong M, McDearmid JR et al. (2002) Development of the locomotor network in zebrafish. Prog Neurobiol 68: 85-111. doi:10.1016/S0301-0082(02)00075-8. PubMed: 12450489. 12450489 10.1016/s0301-0082(02)00075-8

[65] Brustein E, Saint-Amant L, Buss RR, Chong M, McDearmid JR et al. (2003) Steps during the development of the zebrafish locomotor network. J Physiol Paris 97: 77-86. doi:10.1016/j.jphysparis.2003.10.009. PubMed: 14706693. 14706693 10.1016/j.jphysparis.2003.10.009

[66] Buss RR, Drapeau P (2001) Synaptic drive to motoneurons during fictive swimming in the developing zebrafish. J Neurophysiol 86: 197-210. PubMed: 11431502. 11431502 10.1152/jn.2001.86.1.197

[67] Thirumalai V, Cline HT (2008) Endogenous dopamine suppresses initiation of swimming in prefeeding zebrafish larvae. J Neurophysiol 100: 1635-1648. doi:10.1152/jn.90568.2008. PubMed: 18562547. 18562547 10.1152/jn.90568.2008 PMC2544474

[68] Xi Y, Ryan J, Noble S, Yu M, Yilbas AE et al. (2010) Impaired dopaminergic neuron development and locomotor function in zebrafish with loss of pink1 function. Eur J Neurosci 31: 623-633. doi:10.1111/j.1460-9568.2010.07091.x. PubMed: 20141529. 20141529 10.1111/j.1460-9568.2010.07091.x

[69] Brustein E, Chong M, Holmqvist B, Drapeau P (2003) Serotonin patterns locomotor network activity in the developing zebrafish by modulating quiescent periods. J Neurobiol 57: 303-322. doi:10.1002/neu.10292. PubMed: 14608665. 14608665 10.1002/neu.10292

[70] Brustein E, Drapeau P (2005) Serotoninergic modulation of chloride homeostasis during maturation of the locomotor network in zebrafish. J Neurosci Off J Soc Neurosci 25: 10607-10616. doi:10.1523/JNEUROSCI.2017-05.2005. PubMed: 16291933. 10.1523/JNEUROSCI.2017-05.2005 PMC6725851 16291933

[71] Missale C, Nash SR, Robinson SW, Jaber M, Caron MG (1998) Dopamine receptors: from structure to function. Physiol Rev 78: 189-225. PubMed: 9457173. 9457173 10.1152/physrev.1998.78.1.189

[72] Boehmler W, Carr T, Thisse C, Thisse B, Canfield VA et al. (2007) D4 Dopamine receptor genes of zebrafish and effects of the antipsychotic clozapine on larval swimming behaviour. Genes Brain Behav 6: 155-166. doi:10.1111/j.1601-183X.2006.00243.x. PubMed: 16764679. 16764679 10.1111/j.1601-183X.2006.00243.x

[73] Boehmler W, Obrecht-Pflumio S, Canfield V, Thisse C, Thisse B et al. (2004) Evolution and expression of D2 and D3 dopamine receptor genes in zebrafish. Dev Dynam Off Publ American Association Of Anatomists 230: 481-493. doi:10.1002/dvdy.20075. PubMed: 15188433. 10.1002/dvdy.20075 15188433

[74] Bundschuh ST, Zhu P, Schärer YP, Friedrich RW (2012) Dopaminergic modulation of mitral cells and odor responses in the zebrafish olfactory bulb. J Neurosci Off J Soc Neurosci 32: 6830-6840. doi:10.1523/JNEUROSCI.6026-11.2012. PubMed: 22593052. 10.1523/JNEUROSCI.6026-11.2012 PMC6622199 22593052

[75] Easter SS Jr., Nicola GN (1996) The development of vision in the zebrafish (Danio rerio). Dev Biol 180: 646-663. doi:10.1006/dbio.1996.0335. PubMed: 8954734. 8954734 10.1006/dbio.1996.0335

[76] Easter SS Jr., Nicola GN (1997) The development of eye movements in the zebrafish (Danio rerio). Dev Psychobiol 31: 267-276. doi:10.1002/(SICI)1098-2302(199712)31:4. PubMed: 9413674. 9413674 10.1002/(sici)1098-2302(199712)31:4<267::aid-dev4>3.0.co;2-p

[77] Whitlock KE, Westerfield M (2000) The olfactory placodes of the zebrafish form by convergence of cellular fields at the edge of the neural plate. Development 127: 3645-3653. PubMed: 10934010. 10934010 10.1242/dev.127.17.3645

[78] Kimmel CB, Ballard WW, Kimmel SR, Ullmann B, Schilling TF (1995) Stages of embryonic development of the zebrafish. Dev Dynam Off Publ American Association Of Anatomists 203: 253-310. doi:10.1002/aja.1002030302. 10.1002/aja.1002030302 8589427

[79] Thisse C, Thisse B (2008) High-resolution in situ hybridization to whole-mount zebrafish embryos. Nat Protoc 3: 59-69. doi:10.1038/nnano.2008.25. PubMed: 18193022. 18193022 10.1038/nprot.2007.514

