# Supplementary material for: Aromatic L-Amino Acid Decarboxylase (AADC) Is Crucial for Brain Development and Motor Functions
Source: PLoS One. 2013 Aug 5;8(8):e71741. doi: 10.1371/journal.pone.0071741 (PMC3734303; doi:10.1371/journal.pone.0071741)
Supplement: Table S1 — Sequences of morpholino oligonucleotides and anti-sense probes for whole-mount in situ hybridization analysis used in this study. (PDF) [file pone.0071741.s001.pdf]

Table S1. Sequences of morpholino oligonucleotides and anti-sense probes for whole-mount in situ hybridization analysis used in this study.

Morpholino oligonucleotides	Sequence	
ddc tMO1	5'-TCTCCGAAACTCTGCGGCATCCATC-3'	
ddc tMO2	5'-TGCGGCATCCATCTTTTAGTAGTGT-3'	
Standard MO	5'-CCTCTTACCTCACTTTACAATTTATA-3'	
Antisense probes	NCBI Accession #	Probe region
ddc	NM_213342.1	111…1553
th	NM_131149.1	270…1080
huC	U62018.1	1…1119
