# Supplementary material for: Aromatic L-Amino Acid Decarboxylase (AADC) Is Crucial for Brain Development and Motor Functions
Source: PLoS One. 2013 Aug 5;8(8):e71741. doi: 10.1371/journal.pone.0071741 (PMC3734303; doi:10.1371/journal.pone.0071741)
Supplement: Figure S1 — Efficiency testing of ddc translational blocking MOs. (A) A 303 bp partial sequence of ddc containing tMO1 or tMO2 targeting site (purple region) was inserted into a pCS2+ vector containing CMV promoter (brown region) and GFP (green region) sequences. The binding sequences for ddc tMO1 and tMO2 are shown below the construct map. (B) Embryos were co-injected with designated MO (5 ng) and MO efficiency testing plasmid (100 pg), examined by epifluorescent microscope under bright and dark fields. Representative superimposed images are shown. (C) Percentages of embryos showing GFP in different treatments are shown. Plasmid injected only group, n=106; tMO1,n=103; tMO2, N=64. * p <0.001. (PDF) [file pone.0071741.s002.pdf]

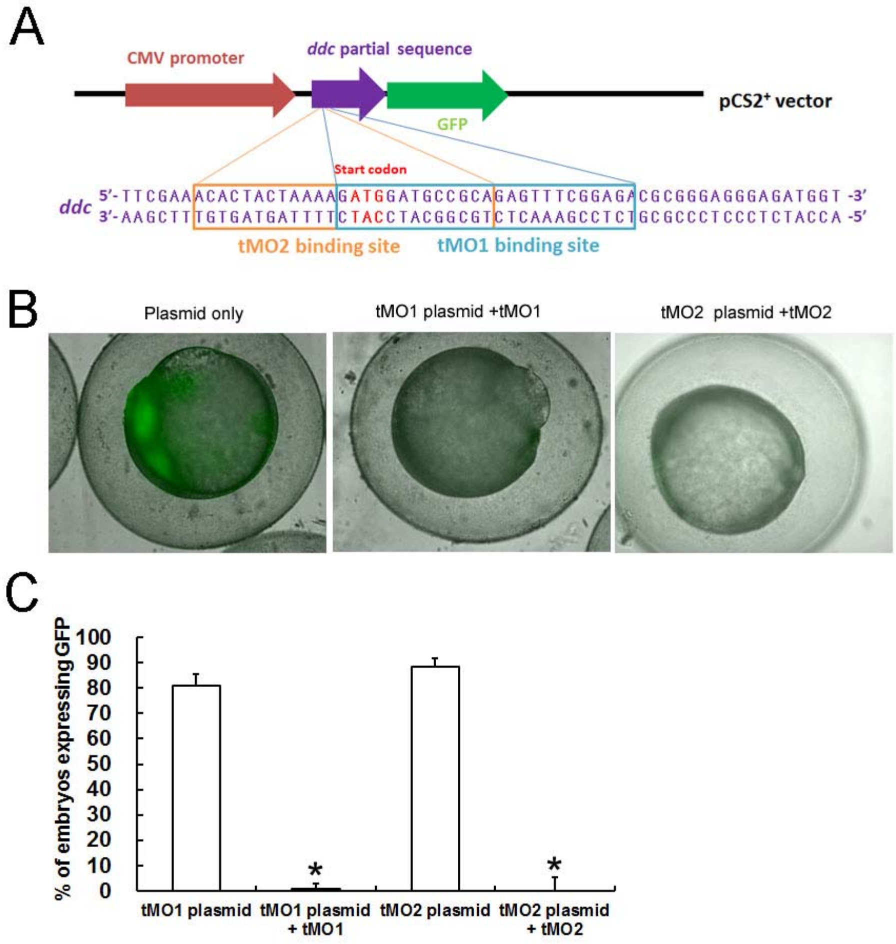

Fig. S1. Efficiency testing of *ddc* translational blocking MOs. (A) A 303 bp partial sequence of *ddc* containing tMO1 or tMO2 targeting site (purple region) was inserted into a pCS2$^+$ vector containing CMV promoter (brown region) and GFP (green region) sequences. The binding sequences for *ddc* tMO1 and tMO2 are shown below the construct map. (B) Embryos were co-injected with designated MO (5 ng) and MO efficiency testing plasmid (100 pg), examined by epifluorescent microscope under bright and dark fields. Representative superimposed images are shown. (C) Percentages of embryos showing GFP in different treatments are shown. Plasmid injected only group, n=106; tMO1,n=103; tMO2, N=64. * p <0.001.
