# Supplementary material for: Aromatic L-Amino Acid Decarboxylase (AADC) Is Crucial for Brain Development and Motor Functions
Source: PLoS One. 2013 Aug 5;8(8):e71741. doi: 10.1371/journal.pone.0071741 (PMC3734303; doi:10.1371/journal.pone.0071741)
Supplement: Figure S2 — ddc MO caused reduction in brain size as examined by whole-mount in situ hybridization against huc. Embryos were treated as indicated and subjected to whole-mount is situ hybridization against huc. (PDF) [file pone.0071741.s003.pdf]

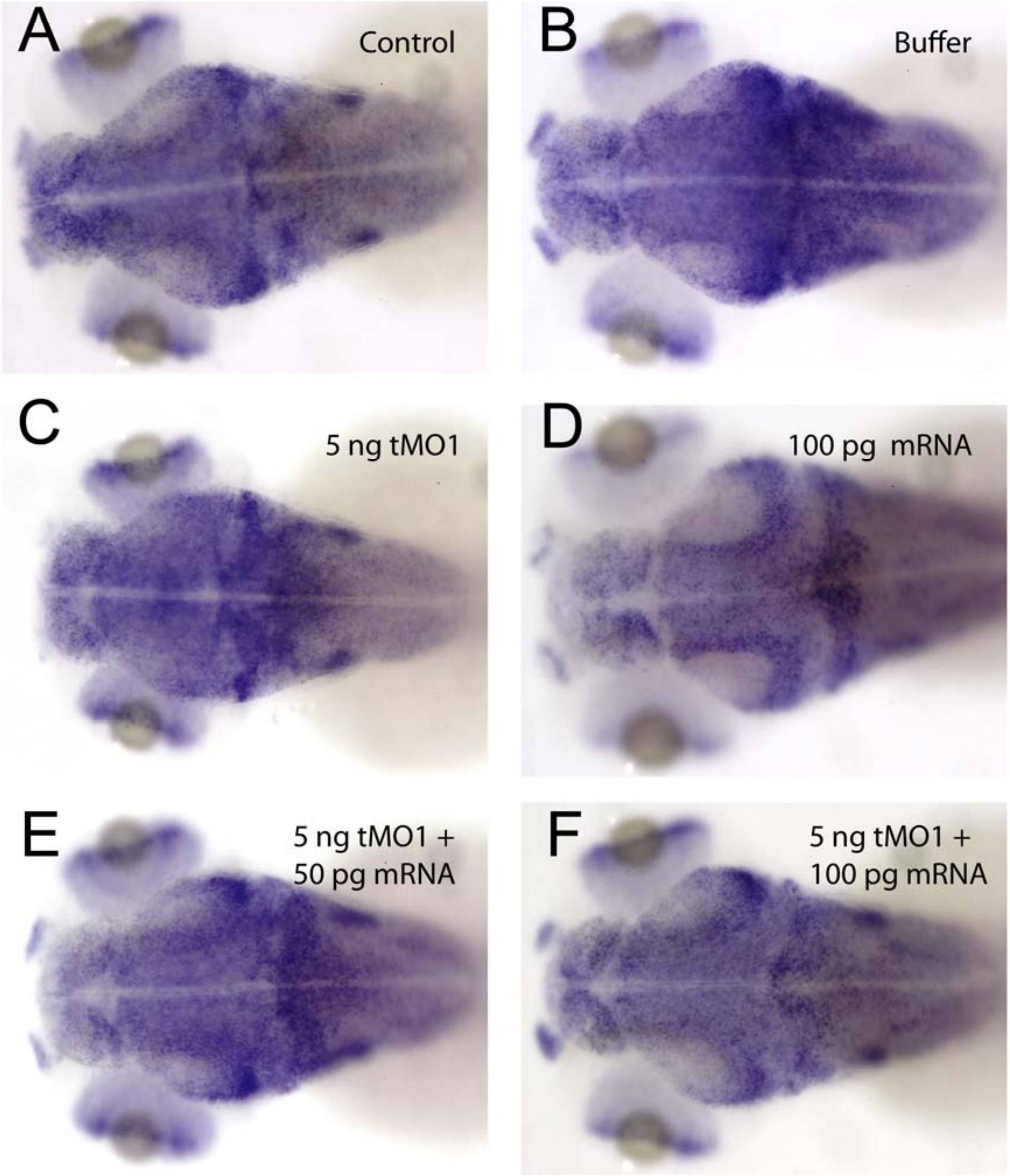

Fig. S2 *ddc* MO caused reduction in brain size as examined by whole-mount *in situ* hybridization against *huc*. Embryos were treated as indicated and subjected to whole-mount is situ hybridization against *huc*.
